# Flexible Soft-Printed Polymer Films with Tunable Plasmonic Properties

**DOI:** 10.1021/acsmaterialsau.3c00023

**Published:** 2023-09-05

**Authors:** Aleksei Solomonov, Anna Kozell, Alexander B. Tesler, Iddo Pinkas, Seth Walensky, Ulyana Shimanovich

**Affiliations:** † Department of Molecular Chemistry and Materials Science, Weizmann Institute of Science, 76100, 234 Herzl Street, Rehovot, Israel; ‡ Department of Materials Science and Engineering, Friedrich-Alexander-Universität Erlangen-Nürnberg, Martensstrasse 7, Erlangen, 91056, Germany; § Chemical Research Support, Weizmann Institute of Science, 76100, 234 Herzl Street, Rehovot, Israel

**Keywords:** gold nanostructures and nanoparticles, plasmonics, solid polymer films, soft printing, tape lithography, SERS

## Abstract

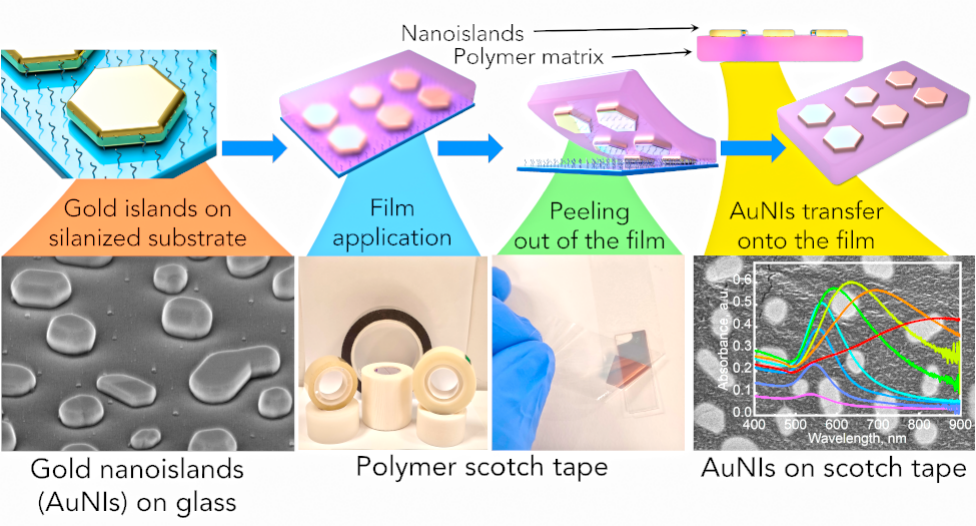

Noble metal nanoparticles (NPs) and particularly gold (Au) have become emerging materials in recent decades due to their exceptional optical properties, such as localized surface plasmons. Although multiple and relatively simple protocols have been developed for AuNP synthesis, the functionalization of solid surfaces composed of soft matter with AuNPs often requires complex and multistep processes. Here we developed a facile approach for functionalizing soft adhesive flexible films with plasmonic AuNPs. The synthetic route is based on preparing Au nanoislands (AuNI) (ca. 2–300 nm) on a glass substrate followed by hydrophobization of the functionalized surface, which in turn, allows efficient transfer of AuNIs to flexible adhesive films via soft-printing tape lithography. Here we show that the AuNI structure remained intact after the hydrophobization and soft-printing procedures. The AuNI-functionalized flexible films were characterized by various techniques, revealing unique characteristics such as tunable localized plasmon resonance and Raman enhancement factors beneficial for chemical and biological sensing applications.

## Introduction

When dimensions of noble metals—including gold (Au), silver (Ag), copper (Cu), and others—reduce to a less than or comparable wavelength of the incident light, they exhibit distinct optical characteristics derived from the excitation of localized surface plasmon (LSP) polaritons in/or close to, the visible spectral range.^1^ LSPs are collective charge density oscillations confined within the final volume of a metallic nanoparticle (NP). When the frequency of incident light is equal to the plasma frequency of the free electrons, resonance occurs, resulting in strong light scattering, the appearance of intense LSP absorption bands, and the enhancement of the local electromagnetic field near the metal surface.^2^ The characteristics of this resonance are affected by the size, shape, and origin of the nanostructures, as well as plasmon coupling effect in the case of NP assembly.^3^ Furthermore, the wavelength and extinction intensity of the localized surface plasmon resonance (LSPR) band are sensitive to variations in the dielectric properties of the surrounding environment.^4^ Such changes near the NP surface can be induced, for instance, by binding molecules to the metal structures, displacing air by solvent,^5^ or changes in the oxidation state of the substrate.^6^ The latter forms the basis for applying LSPR systems as optical sensors^7,8^ and transducers for chemical and biological sensing.^4^

Tuning the wavelength of the LSPR band is vital for applications such as photothermal therapy,^9^ plasmon-enhanced luminescence,^10^ optical imaging, labeling of biological systems,^11^ photo- and photoelectrocatalysis.^12,13^ The LSP band position can be tuned through (*i*) the nanostructure shape such as rod-like nanostructures that display transverse and longitudinal SP bands,^14^ (*ii*) the nanoparticle size,^3,15^ and (*iii*) their nature, *i.e*., single- or multicomponent metal NPs (*e.g.*, Au, Ag, and/or Cu).^16,17^

There are various ways to synthesize noble metal nanostructures;^18,19^ however, as optical transducers, they can be used either as free-standing NPs dispersed in solution or deposited as a thin metal film on solid transparent substrates such as glass or quartz. Such substrates can be obtained by either lithographic methods that provide good control over the particle shape, dimensions, and array design but are not easily scalable^20−25^ or random ensembles of evaporated metal nanoislands with good control over the particle dimensions, composition, and scalability.^26,27^ In the latter case, metal (*e.g.*, Au) island films, obtained by high vacuum physical vapor deposition techniques, are subjected to postdeposition thermal treatment. Such annealing of ultrathin Au films promotes solid-state dewetting and the coalescence of the metal film triggering the formation of well-defined single-crystalline Au nanoislands and increasing the interisland separation, resulting in a distinct sharp LSPR band with a tunable spectral window.^1,3,28^

Evaporation of thin Au films, followed by high-temperature annealing, is a convenient approach to obtain uniform coverage of substrate surfaces by structures such as nanoparticles (AuNPs) or nanoislands (AuNIs) on solid substrates with well-defined optical and morphological properties that uniformly fill desired surfaces.^29,30^

Various applications require the incorporation of plasmonic NPs not just onto/into solid inorganic materials but organic polymers (e.g., PMMA), biopolymers (proteins),^31−33^ and polysaccharides to form the so-called plasmonic nanocomposites,^34^ that provide additional functionality such as substrate flexibility.^35,36^ Fabrication of metal NPs in a polymer matrix is challenging since NPs usually aggregate alternating their SPR characteristics. In recent years, controlled assembly of NPs directly on/in polymeric substrates has been proposed based on the self-assembly of NPs from solution.^34,37,38^ Growing Au NPs on a flexible substrate enables simple mechanical control of the plasmonic coupling via its stretching/shrinkage.^39^ Plasmon-coupled AuNPs incorporated in flexible stretched shape-memory polymers were demonstrated for mechanical and thermal sensing,^40^ and opto-mechanic devices.^41,42^

To speed up the process, transfer methods of tape nanolithography were proposed.^43,44^ In this approach, NPs are transferred to adhesive tape via a soft-printing mechanism. The nanopatterns are first formed on a donor substrate and, after application of adhesive tape, are automatically transferred to the deposited tape material. One of the advantages of such flexible soft-printed NP-comprised substrates, particularly for sensing applications, is that they can catch target analytes directly from a nonflat surface. On the other hand, as part of an integrated circuit, the NP-based flexible substrates could be utilized in wearable electronics and flexible optical materials, to name but a few. A soft-printing approach is attractive, since it preserves the pattern of NPs originally formed on a solid-state substrate.

However, the existing approaches rely on site specific detachment of NPs from template substrate via introducing either voids or weak interactions between the templating substrate and NPs. Such approaches often suffer from inconsistency in NPs transferring yield and uneven distribution of NPs on the surface of factionalized (NP acceptor) soft and flexible films. Recently it was demonstrated that Ag-decorated adhesive tapes were used as flexible SERS-active substrates for pesticide detection on fruit skin,^45,46^ and also Ag-based adhesive tape was applied for near-infrared photodetection.^47^ Decoration of adhesive tape with conductive NPs leads to increased conductivity of the tape.^48^ The tape lithography was used to replace several ion beam milling steps and can create inverse plasmonic structures such as nanoholes and nanoslits arrays.^49^

To address existing technological challenges, here we designed and developed a facile soft-printing approach to efficiently functionalize flexible polymeric materials with a series of AuNIs with tunable plasmonic characteristics. The AuNIs with a tunable LSPR band position were first prepared on glass substrates (template), followed by their high-temperature annealing in air. The annealing conditions were optimized for (*i*) complete dewetting and coalescence of ultrathin Au films into well-defined NIs and (*ii*) stabilization of the formed NIs on the glass surface for further chemical functionalization by fluorinated silanes. The latter is crucial for the efficient transfer of AuNIs from the glass substrate to the polymeric tape material. The developed approach has been equally applied to various types of transparent and reflective tapes, such as Scotch, Kapton, and Mylar adhesive tapes as well as Durapore (silk-like fabric), Transpore (polyethylene), and Micropore (viscose) medical adhesive tapes. The developed process is highly efficient and low-cost. We envision that the proposed method will be of great interest in the development of advanced flexible-polymer-based optical and functional materials.

## Results and Discussion

### Preparation and Soft-Print of AuNIs

In comparison to the solvent-based synthesis of NPs, the described approach has substantial advantages such as the following: (1) it is simple, reliable, and predictable; (2) it allows one to prepare particles from less than 1 nm to >1 μm in diameter to continuous Au films with controllable surface coverage by simply varying the nominal Au film thickness, and the number of evaporation/annealing steps;^29^ (3) it allows the fabrication of Au nanostructures that are typically difficult to obtain in solution, specifically relatively large particles with variable crystallinity; (4) it forms uniform random NP arrays with controllable LSPR characteristics resulting in tunable spectral selectivity; (5) AuNIs are free from any capping molecules/agents/surfactants and are ready for further structural and surface modifications; (6) AuNIs exhibit exceptional stability on glass substrates without any buffer layers. Furthermore, AuNIs prepared directly on glass are stable in pure or mixed solvents and biological buffers.^50−52^ The localized surface plasmon resonance (LSPR) peak of AuNIs is well-pronounced, with the narrow full width at half-maximum (fwhm),^3^ and a uniform distribution of AuNIs over a large area.

**Figure 1:**
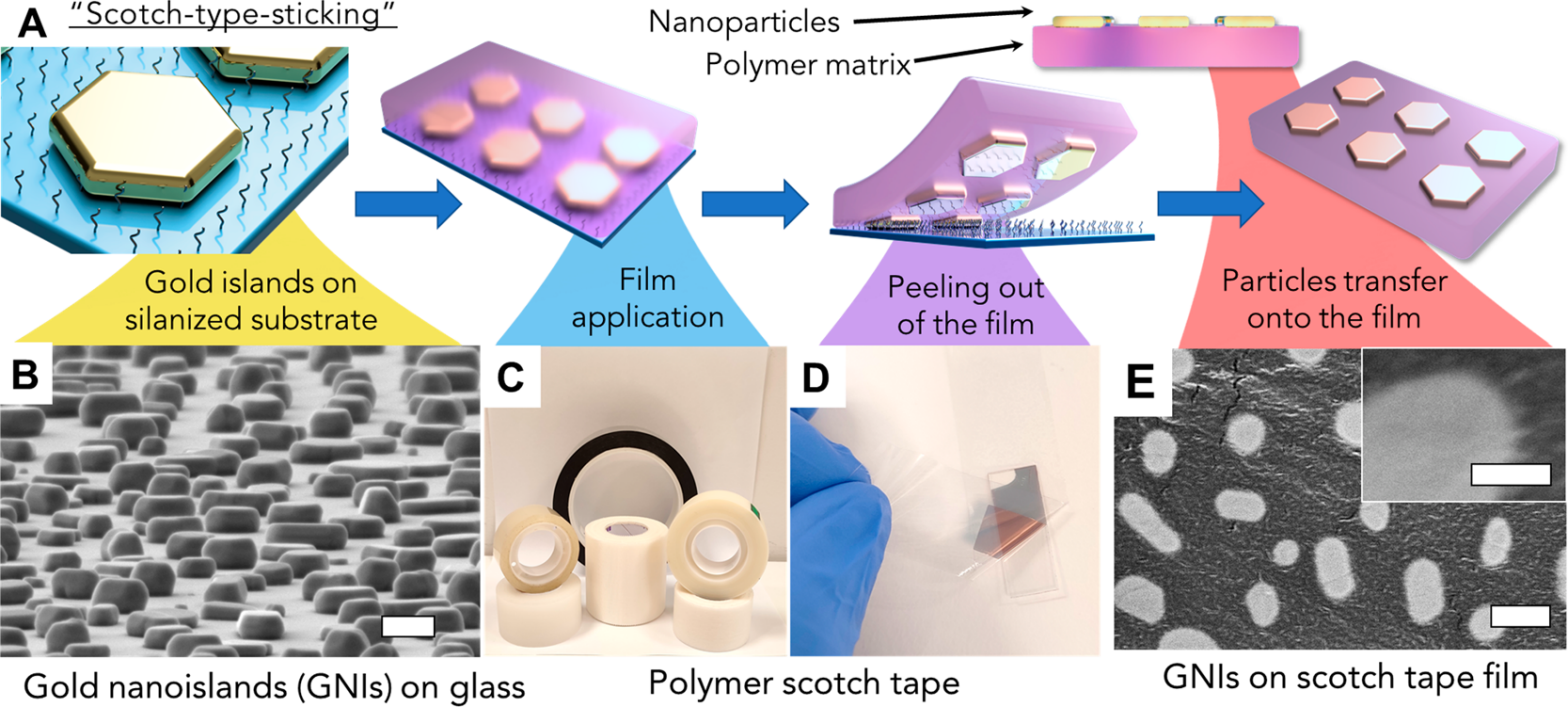
A scotch tape is soft-printed with plasmonic AuNIs. (a) Schematic representation of polymer films templated with AuNIs by embedding them into the film using the “scotch-tape” sticking method. (b) FE-HRSEM (tilted view) of AuNIs on glass formed by Au film evaporation (15 nm nominal thickness) and annealing. (c) Digital image of the scotch film tape used in this study as an example of a synthetic polymer and (d) the tape in the process of AuNI transfer from the glass substrate. (e) Scotch tape film with transferred AuNIs (15 nm nominal thickness). The inset image shows an enlarged area of the film’s morphology. The scale bars for the FE-HRSEM images are (b) 400 nm, (e) 200 nm, and 100 nm for the inset image.

Figure 1a represents schematically the procedure to form gold (Au) nanoislands (AuNIs) on the flexible substrates. More specifically, ultrathin Au films were prepared by electron beam (e-beam) evaporation of Au on borosilicate glass coverslips with a surface roughness of *ca*. 1 nm (Figure S1). The nominal (mass) Au thicknesses varied from 1 to 15 nm with a step size of 2 nm. The as-evaporated Au films on the glass slide appeared in pale red, greenish-blue, and greenish-yellow and have complex shapes (Figure S2a, Figure S3). The as-deposited Au films demonstrate a broad LSPR band in the visible and near-IR spectral regions ranging from ca. 532 nm to >900 nm by an LSPR band maximum (Figure S2b). Note that the percolation threshold for ultrathin Au films deposited on glass substrates is ∼8 nm; i.e., the as-deposited Au films have a depercolated structure below this thickness, whereas beyond this thickness, the films appear as semicontinuous (Figure S3).^3^ The latter is important since the solid-state dewetting/coalescence processes are greatly influenced by the initial film morphology, while percolated films form larger and higher aspect ratio Au islands under the application of high-temperature annealing in air.^28^ Hence, the evaporation thickness range was subdivided into two regions based on the nominal Au thickness: (*i*) 1–7 nm, *i.e*., small spherical-like shaped AuNIs (*type I*), and (*ii*) 9–15 nm, *i.e*., large truncated ellipsoid-like-shaped AuNIs (*type II*). It is well-known that the LSPR band position is highly sensitive to the NI size and shape; therefore, this range of Au thicknesses covers the visible spectral range almost entirely, as shown below.

The as-deposited ultrathin Au films were exposed to high-temperature annealing in air (Figure S1). The annealing triggers a coalescence process in *type I* films, forming predominantly well-defined round-shaped or semispherical AuNIs, whereas, in *type II* films, the solid-state dewetting process occurs first followed by coalescence and recrystallization of depercolated islands; thus, forming large, well-faceted truncated ellipsoid-shaped AuNIs with a clear crystalline structure (Figure 1b and Figure S4).^3^ It was previously demonstrated that the coalescence process of the initially depercolated Au films on glass occurs within a few minutes at 550 °C, whereas with semicontinuous films, the process proceeds for longer times.^28^ However, this annealing temperature approaches the glass transition temperature of borosilicate glass (*T*_g_ ≈ 557 °C) leading to the partial embedding of AuNIs and, consequently, their substantial stabilization.^1,3,28,50,53,54^ The latter is extremely important in the case where AuNIs are applied directly in chemical and biological sensing applications, while in the case of the soft-print process it may substantially retard the process of AuNI delamination. Therefore, in this study, all as-deposited Au substrates were annealed at 450 °C, i.e., much beyond the substrate glass transition, for 20 h in air to ensure (*i*) completeness of AuNI coalescence/dewetting processes forming well-faceted crystalline AuNIs with narrow LSPR band, and (*ii*) to avoid embedding into the substrate achieving unhindered delamination of AuNIs from the glass and their further transfer onto the soft polymeric films.

Statistical analysis of AuNIs (Figure S5 and Figure S6) showed rather uniform distribution across all the thicknesses with average size varies from *ca*. 6.9 to 38.1 nm in average diameter corresponding to *type I* films, and 85.1 to 232 nm corresponding to *type II* films, respectively (Figure S6a). Note that the average particle diameter rapidly increases during the transition from 7 to 9 nm in nominal Au thickness (Figure S6b). The AuNIs density tends to decrease with an increase in nominal Au thickness (Figure S6d), while the surface coverage reaches the maximum value for thicknesses of 3–7 nm (*ca*. 35%) and then decreases as the AuNI size and height increase; i.e., Au tends to dewet/coalesce into large islands with a lower volume-to-area ratio and reaches the minimum of 18.4% for 15 nm-thick Au films (Figure S6e). The height of AuNIs increases gradually from 7.25 to 31.8–53.2 nm for *type I* and intermittently increases while switching to AuNIs of *type II* reaching 72.5 to ∼200 nm in its maximum for 15 nm-thick films (Figure S7, Figure S8). The latter is attributed to the different mechanisms of dewetting\coalescence that occur in *type I* and *type II* Au films.^28^

One of the major challenges in the soft-printing process of AuNIs via traditional methods is the strong attachment of soft material to a solid substrate. Moreover, during the lifting-off process, the soft material can be damaged due to either fragility, strong attachment to a substrate, the inability to trap AuNIs, and/or a combination of both. To address the above-mentioned technological challenges, we applied selective silanization of glass with the annealed AuNIs, to reduce its surface energy, *i.e*., to increase the substrate’s hydrophobicity.

Figure S1, Figure S9, and Figure S10 present schematically the silanization approach, in which AuNIs (Figure 1b) on the silanized glass substrates are exposed to scotch tape (Figure 1c), followed by peeling it off from the glass together with trapped AuNIs (Figure 1d,e and Video S1), similar to the approach the graphene was first obtained in the Nobel Prize awarded works.^55,56^ After the silanization process, the substrates demonstrate identical morphology of the AuNIs distribution (Figure S11, Figure S12, and Figure S13). The as-soft-printed AuNIs are selectively sited on the polymeric film reproducing entirely the original pattern as initially obtained on the glass substrate. The AuNIs-tape films are light and mechanically robust, are easily handled, twisted, and bent (Figure S14a,b**)**, stretched, exposed to pressure, applied on the skin (Figure S14c), and reproduce fingerprint texture (Figure S14d–f).

### Morphological Analysis of the AuNIs: Functionalized Adhesive Films

**Figure 2:**
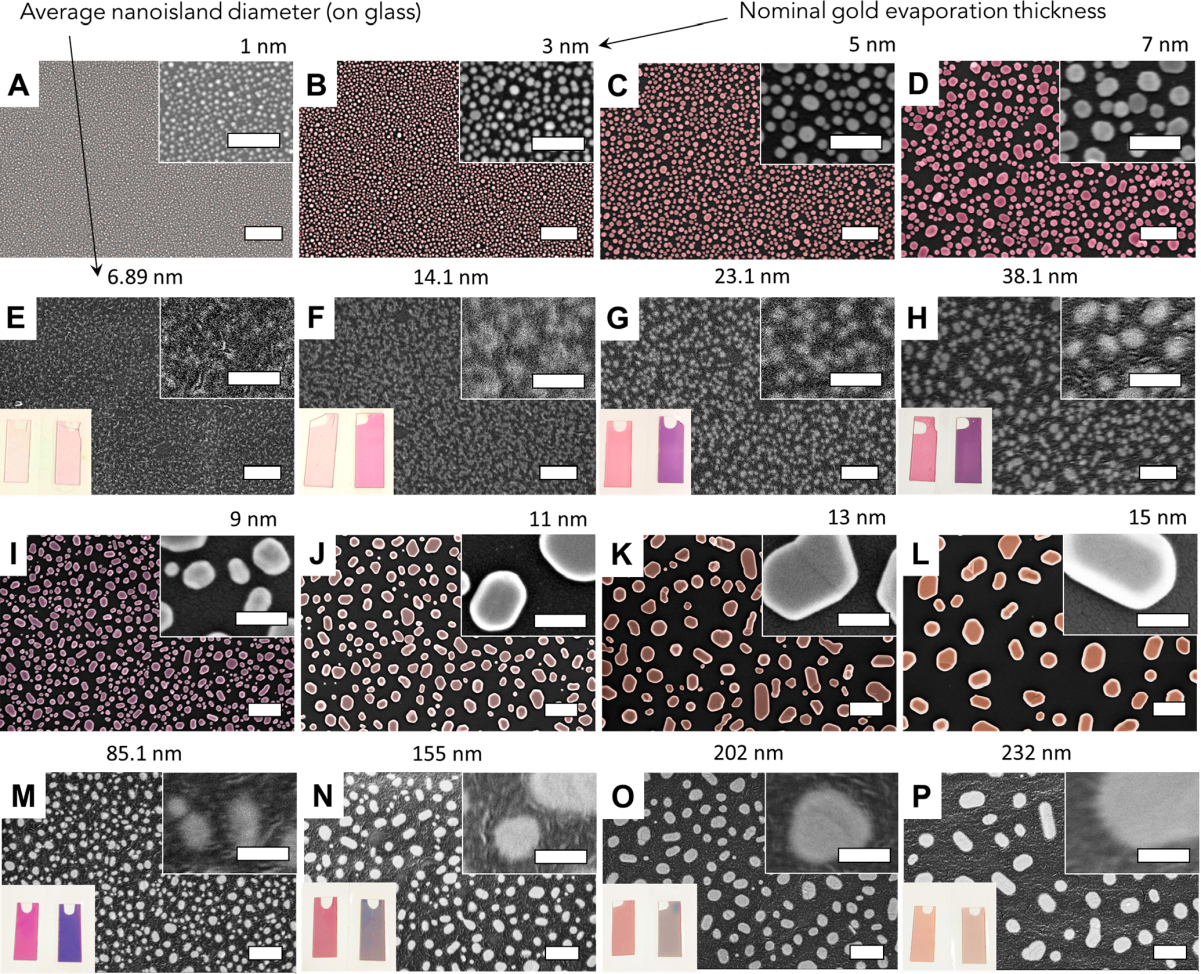
FE-HRSEM images of AuNIs on different substrates. Artificially colored FE-HRSEM of AuNIs formed by gold film evaporation followed by annealing at 450 °C the different nominal thicknesses of Au (a, 1 nm; b, 3 nm; c, 5 nm; d, 7 nm; i, 9 nm; j, 11 nm; k, 13 nm; l, 15 nm), insets, detailed images of the nanostructured gold surface and respective FE-HRSEM of the scotch tape films (e–h and m–p) with AuNIs (nominal gold evaporation thickness: e, 1 nm; f, 3 nm; g, 5 nm; h, 7 nm; m, 9 nm; n, 11 nm; o, 13 nm; p, 15 nm), left bottom insets–digital comparison images of AuNIs on glass slide before (left) and after (right) scotch tape sticking. The top right insets for parts a–p are detailed FE-HRSEM images taken with high magnification. Scalebar for FE-HRSEM images a–h is 200 nm, for parts i–p is 400 nm, and for all the insets is 100 nm.

Initially, the soft-printing process was applied to Scotch brand adhesive tape. The morphology of AuNIs was characterized by field-emission high-resolution scanning electron microscopy (FE-HRSEM) imaging (Figure 2). FE-HRSEM images obtained by the energy-selective backscattered electron detector confirmed the uniform distribution of AuNIs on the Scotch brand tape (Figure S15a), whereas images from the secondary electron detector display a rough structure of the film with AuNIs partially embedded into the tape structure (Figure S15b). As shown, the AuNI pattern on the tape reproduces the entire structural organization of AuNIs formed on the glass, validating the high efficiency of the soft-printing process. Statistical analysis of the AuNIs distribution on the Scotch brand tape shows AuNI allocation patterns across all thicknesses similar to that obtained on glass (Figure S16). The major (maximal) diameter and aspect ratio of the AuNIs are preserved after the soft-printing process (Figure S17).

Note that FE-HRSEM images were taken from the adhesive side of the film that comprises AuNIs to ensure that the fringe of AuNIs that were in contact with the glass is observed. This fringe is flat and similar to the top [111]-facet of bare AuNIs (Figure 2). The tape covers the AuNIs in such a way that the back facet (the facet that was previously in contact with glass) is only visible; i.e., the top facet of AuNI is now in contact with the adhesive film, and side edges are partially exposed to the film (Figure 2e–h and Figure 2m–p). The latter could be controlled by adjusting the applied mechanical force (pressure) during AuNI transfer (see also Figure S18).

The suggested soft-printing process is not limited to scotch tape but may be applied to a variety of adhesive polymeric materials. Here, carbon and metallic Mylar (reflective and conductive) tapes were used to trap AuNIs (Figure S18). Metallic Mylar tape, which possesses reflective and conductive properties, was applied to transfer AuNIs demonstrating comparable transfer efficiency (Figure S18 and Figure S19). FE-HRSEM images confirmed a transfer efficiency similar to that found on the 3M Scotch tape, demonstrating that the soft-printing process of AuNIs is uniform for any adhesive polymeric films (Figure S20).

The soft-printing process efficiency of AuNIs is strictly dependent on several parameters. AuNI trapping on the Scotch brand tape depends strongly on its attachment. If the scotch film was well-attached to a slide without air bubbles, the glass slide appears completely transparent after the soft-printing process (Figure S21a,b). The spectra of the glass substrates were measured, and the LSPR band was not observed (Figure S21c,d), whereas solitary AuNIs can be spotted typically on the glass edges (Figure S22). Large-scale energy-dispersive X-ray spectroscopy (XEDS) analysis revealed similar spectra of the glass slide with AuNIs before and after the silanization procedure, whereas after the soft-printing process the XEDS measurements reproduce the spectrum of bare glass only (Figure S23). The FE-HRSEM, UV–vis, and XEDS analysis indicate that the proposed soft-printing approach is efficient and robust to transfer AuNIs prepared on the glass substrates to the flexible polymeric matrices. To further improve the soft-printing process, the following alterations may be considered: (*i*) stronger adhesive tape can be exploited, (*ii*) more force could be applied during the transfer procedure, (*iii*) pretreatment of the annealed AuNI glass slide with UV-ozone before the silanization process, and (*vi*) the replacement of perfuorooctyltrichlorosilane with milder perfuorooctyltriethoxysilane that led to similar results of the AuNIs transfer to the tape under the same hydrophobization procedure (Figure S24).

### Optical Properties of Bare AuNIs and AuNI-Functionalized Adhesive Films

**Figure 3:**
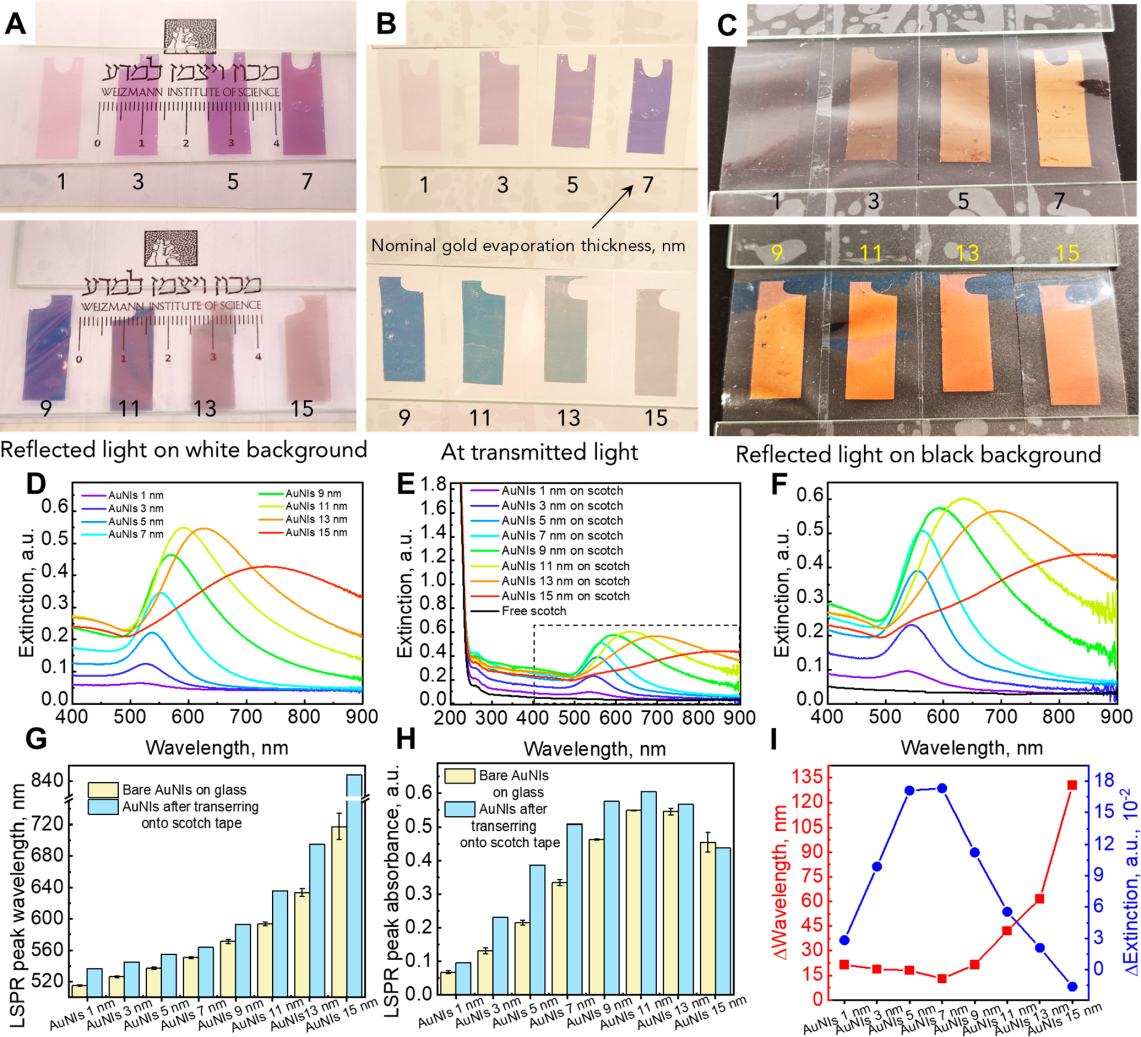
Optical properties of AuNI–scotch tape films. (a–c) Digital images of the scotch tape with embedded AuNIs in reflective mode on (a) a black background, image courtesy of the Weizmann Institute of Science, where the ruler is in cm, (b) on a white background, and (c) in transparent mode. UV–vis spectra of (d) bare AuNIs on a glass slide and (e, f) Scotch films with transferred AuNIs. The LSPR band (g) wavelength and (h) extinction maxima of bare AuNIs on glass (yellow) and free-standing AuNI-scotch tape films (blue) and (i) the calculated LSPR wavelength/extinction shifts for every AuNI thickness.

The optical characteristics of the AuNI-functionalized adhesive films were examined. The obtained films had a uniform color distribution across the entire surface. The color of the substrates changes according to the optical mode, *i.e*., reflectance or transmittance. In the reflectance mode with a white background, the film color appears from pale red to purple for *type I* AuNIs and from violet to yellow for *type II* AuNIs (Figure 3a). In the transmittance mode, the films appear from pale red to purple-violet for *type I* AuNIs, and from blue to green and yellow for *type II* AuNIs (Figure 3b). In the reflectance mode with a black background, all samples appear metallic-gold with increasing intensity from small to large AuNIs (Figure 3c). Yet, there is a slight difference in the color of the same AuNI films deposited either on glass or polymer substrate (Figure 3d–f). This is attributed to the partial embedding of AuNIs into the polymeric matrix, *i.e*., the surrounding environment that has a higher refractive index (*n*_*scotch*_ = ∼ 1.47), whereas on glass, it was exposed mainly to air (*n*_*air*_ = 1). This change is pronounced when comparing bare glass and glass covered by the polymer film with AuNIs (see Figure 2e–h, m–p, insets). Here, AuNIs on bare glass display the LSPR band position in the range of *ca*. 520–725 nm wavelengths (Figure 3d), whereas the same AuNIs on Scotch brand tape show a redshift to *ca*. 540–850 nm wavelengths covering the green-near IR regions (Figure 3e,f). Note that the scotch tape is transparent in UV-A and UV-B (280–400 nm), and partially transparent in UV-C (240–280 nm) spectral regions offering advances in terms of optical transparency relative to glass/quartz.

The interaction of AuNIs with the tape was further characterized in terms of the LSPR band wavelength and absorbance intensity shifts in the UV–vis spectra, as summarized in Figure 3**g-i**. As shown, after embedding AuNIs into the polymer, the LSPR band demonstrates a redshift with an increase in AuNI size (Figure 3**g, i**). For the *type I* films, the LSPR band wavelength redshifts occur around 20 nm corresponding to ∼40 nm/RIU sensitivity, while the *type II* films demonstrate a substantial redshift with a maximum of *ca*. 135 nm for the 15 nm-thick AuNIs, corresponding to ∼300 nm/RIU (Figure 3g,i). At the same time, the extinction intensity increases in *type I* films with an increase in AuNI size while decreasing continuously in *type II* films (Figure 3h,i). These results corroborate our previous findings obtained by the exposure of AuNIs films to solvents with increased refractive indices.^3^

### Evaluation of AuNIs Embedded into the Polymer Matrix via Numerical Modeling

**Figure 4:**
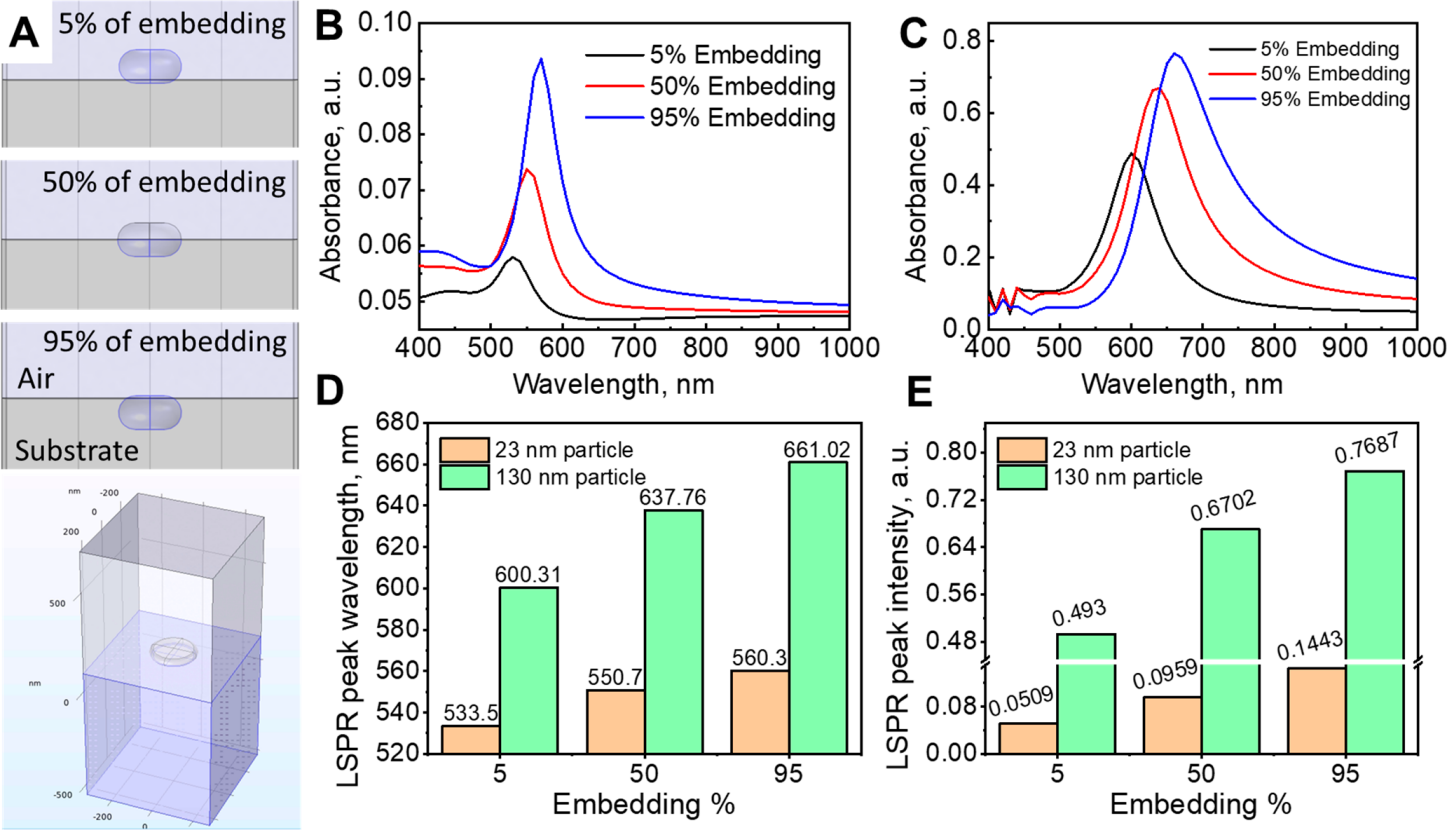
(a) Schematic representation of the simulation geometry of different embedding depths of the AuNIs into a substrate, (b, c) the calculated normalized absorbance spectra of the AuNIs of (b) 23 nm and (c) 130 nm in diameter at different embedding values, and (d, e) the corresponding changes in (d) the LSPR peak wavelength and (e) the LSPR peak intensity of AuNIs embedded at 5, 50, and 95% into the material.

To verify how AuNI embedding onto a polymeric substrate affects the spectral characteristics of the AuNI plasmonic band, computational simulations were applied based on the finite element approach (Figure 4). Two sizes of AuNI were simulated, corresponding to NIs obtained after annealing *ca*. 5 and 11 nm-thick Au layers, i.e., *typeI* and *II* films. The substrate was considered to be a layer that simulates soft (polymer) and hard (glass) material having a refractive index close to *n* = 1.5 in the Vis–NIR spectral range (400–1000 nm). Figure 4a presents cross-sectional images illustrating conditions when 23 nm diameter AuNI is embedded 5, 50, and 95% in a substrate. The 3D image demonstrates the simulation cell of 130 nm in diameter AuNI embedded 50% in a substrate. To emphasize the difference, the shape of the 23 nm in diameter AuNI was approximated as spherical, whereas the one of 130 nm in diameter was approximated as an oblate spheroid with a flat top surface (according to the cross-sectional FE-HRSEM images).

According to simulations, the LSPR peak is red-shifted, and its intensity increases with the degree of embedding. For the 23 nm particles, the relative LSPR shift reaches 17 and 38 nm wavelength redshifts, whereas the embedding degree increases from 5 to 50% for 23 and 130 nm islands, respectively. The LSPR redshift is even more pronounced when AuNIs are embedded at 95%, reaching ca. 25 and 61 nm wavelength shifts for 23 and 130 nm AuNIs, respectively. The LSPR band intensity increases with increasing degrees of embedding.

Simulations revealed that the LSPR peak position of the 5%-embedded AuNIs of both sizes matched well the LSPR band position of AuNIs on glass (Figure 2) before their transfer to soft material. While comparing the experimental data of the LSPR band position of AuNIs after the transfer to scotch tape with simulations, it was found that the band position of simulated spectra corresponds to 40–60% of AuNI embedded into the soft material. Despite an ideal case scenario of AuNI location obtained in the numerical simulations and considering spatial order and shape as well as size uniformity, these results correlate well with the tilted SEM images obtained on the soft-printed AuNIs on Scotch-style tape (see Figure S18), which shows that the AuNIs are embedded at about half of their initial height and correlate with experimental data of peak position. Therefore, the computational simulations model well the experimental data of AuNIs embedded into soft material. These models of partial embedding of AuNIs into a polymeric material with a known refractive index may serve as a plasmonic ruler to develop optical pressure sensors.

### Applications of the AuNI-Embedded Scotch Tape

#### SERS of Rhodamine 6G Adsorbed on Scotch Tape

**Figure 5:**
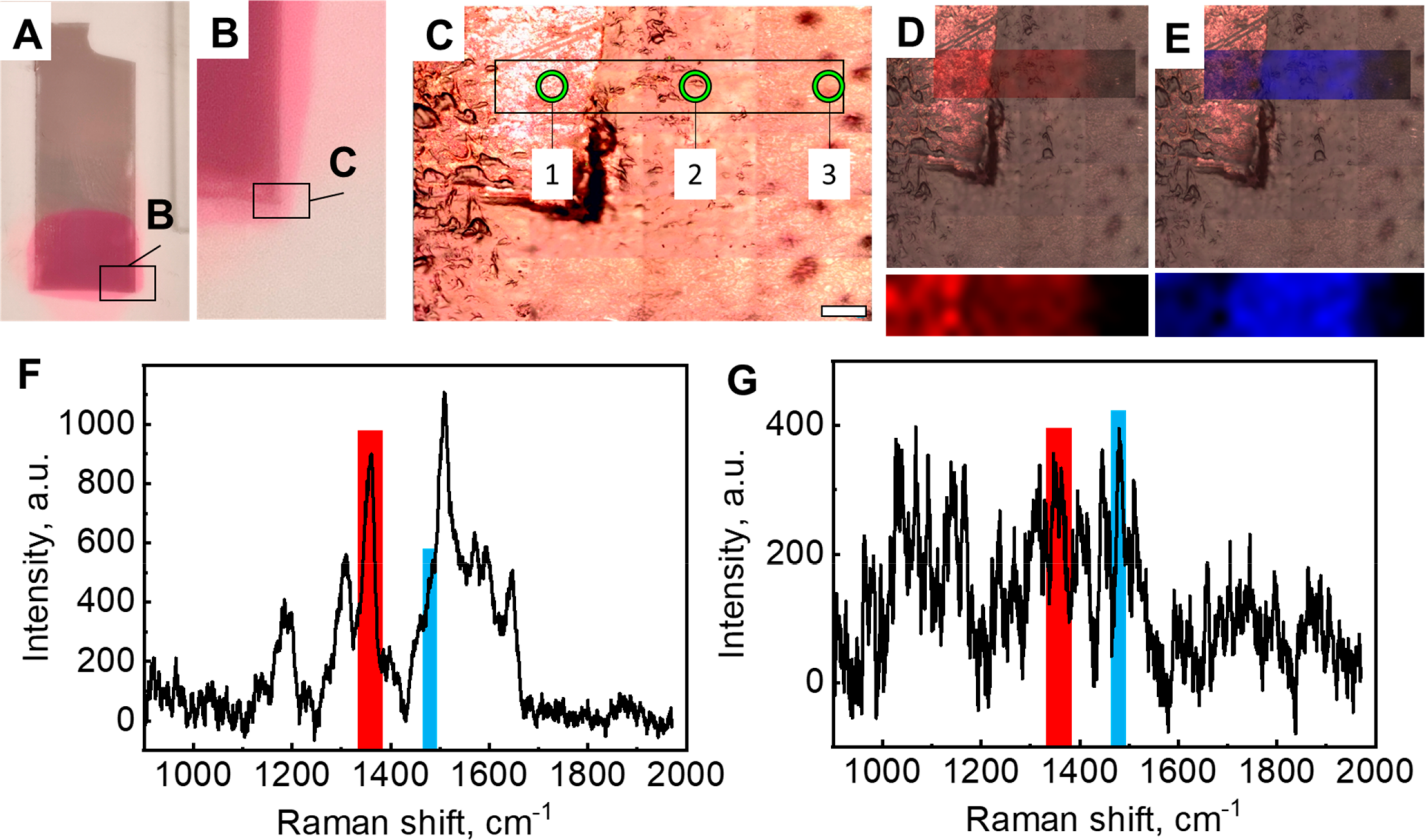
(a, b) Digital images of the scotch tape with soft-printed AuNIs (11 nm of nominal gold evaporation thickness) with adsorbed R6G at the bottom. (c) Microscopy images of the area of the tape taken in the mapping regime showing different areas of the tape: (1) with AuNIs and with adsorbed R6G, (2) with adsorbed R6G without AuNIs, and (3) the area of a pristine tape. Scalebar is 50 μm. Overlaid and separate images of the intensity distribution in the ROI depicted in part c are for Raman spectral ranges (d) from 1335 and 1375 cm^–1^ and (**e**) from 1465 to 1495 cm^–1^. Raman spectra correspond to (f) area 1 and (g) area 2 (see part c). Red and blue lines in parts f and g display Raman regions corresponding to the Raman mode mapped in parts d and e.

The sensing characteristics of flexible plasmonic tapes were tested by the adsorption of Rhodamine 6G (R6G) (Figure 5a,b) for Raman spectroscopy measurements. Figure 5c shows an area displayed in Figure 5b taken in a mosaic regime showing different areas of the region of interest (ROI). The intensity of the red in the ROI of this figure is derived from a region of the Raman spectra that was measured in this region between 1335 and 1375 cm^–1^. The highest intensity was obtained in area 1 of the ROI (see Figure 5c), decreasing in the central part (Figure 5c, area 2), and was not observed on the right-hand side (Figure 5c, area 3). Figure 5f demonstrates a typical spectrum from the region corresponding to area 1 (Figure 5c), showing an intense R6G AuNI-enhanced Raman spectrum. The intensity of the blue color in the ROI is derived from a part of the Raman spectrum measured from this region in the spontaneous (not surface enhanced) Raman shift range from 1465 to 1495 cm^–1^. The highest intensity is in the center of the ROI and a slightly lower intensity is on the left of the ROI, while the right-hand side of the ROI is almost dark. Figure 5e demonstrates a typical Raman map from the region corresponding to area 2 (see Figure 5c) showing a noisy R6G characteristic spectrum, confirming that AuNIs provide surface enhancement of the R6G Raman spectrum in area 1 but not in area 2. Finally, it was impossible to obtain a characteristic spectrum for the furthest right-hand section of the ROI (see Figure 5c, area 3), which has the characteristics of the Scotch brand tape spectrum that under these detection conditions is virtually undetected due to the low signal intensity and high noise (not shown).

#### Multiplasmonic Tape Fabrication

After AuNIs are soft-printed onto the adhesive tape, they remain sticky and are ready for another layer of islands capture (Figure S25a–e). This is because AuNIs cover between 20 to 35% of the tape surface. After the second soft-printing procedure, AuNIs overlap partially (Figure S25f–m). This procedure can be repeated multiple times with different sizes of AuNIs providing an opportunity for multiplasmonic system fabrication on the same substrate (Figure S25f–m).

#### Patterning Ability

Furthermore, we show that the AuNI transfer procedure could be combined with pattern fabrication made from AuNIs using shadow mask evaporation. This can be achieved by applying a mask comprising the desired pattern (Figure S26a,b) on a slide before Au evaporation after the slide was processed as described earlier. This allowed us to form a simple pattern on a slide that was successfully transferred to adhesive tape (Figure S26a–c). Optical microscopy images of the AuNI patterns formed on a glass slide (Figure S27) and after soft-printing to the tape (Figure S28) show a complete AuNI transfer confirming that the soft printing technology of AuNIs can be combined with lithographical approaches. The procedure of shadow masking evaporation can also be replaced by simply drawing on a slide, followed by successful soft-printing (Figure S29).

#### AuNIs Transfer onto Medical Tapes

Next, we expanded the soft-printing AuNI procedure to various types of biocompatible medical/surgical adhesive tapes, namely, 3M Durapore, 3M Micropore, and 3M Transpore, which serve medical purposes (Figure S30a–c) but have different textures, porosities, and adhesive characteristics. The soft-printing approach works well with these adhesive tapes, and AuNIs of both types can be successfully soft-printed to them. After the transfer, the tapes remain sticky and can be attached to the skin (Figure S30d). Optical microscopy (Figure S31, Figure S34, and Figure S37) and FE-HRSEM imaging (Figure S32, Figure S33, Figure S35, Figure S36, Figure S38, and Figure S39) show the high efficiency of AuNI transfer to medical tapes of all types. Such tapes with transferred AuNIs may serve for medical applications such as phototherapy, transdermal Au-supported drug delivery, or as an antibacterial adhesive. Moreover, it is expected that upon transferring AuNIs to a dissolvable tape and combining it with the patterning ability/electric circuits, islands might be potentially transferred to any kind of surface or skin-creating, *e.g.,* tattoo-like medical biomonitoring devices.^57^

### Transfer onto Kapton Polymer Using a Drop-Casting Method

Finally, to demonstrate the universality of the AuNI soft-printing method, AuNIs were transferred to polymeric films prepared by the drop-casting technique. Like soft printing on adhesive tape, AuNIs cannot be transferred onto a flexible substrate without a preliminary glass slide hydrophobization. Here, liquid polyimide (PI2610, HD MicroSystems, USA) was dropped onto the hydrophobic slide with AuNIs and the film was formed by thermal treatment at 65 °C for 2 days in the oven (Figure S40a–d). After the film is separated from the slide, AuNIs are captured entirely in the polymer matrix, i.e., side-selectively transferred to the formed film, comparable to the soft-printing method on adhesive tapes. Fabricated polymeric substrates display intense plasmonic properties, and the FE-HRSEM images show the same structural organization of the AuNIs (Figure S40e–p).

## Conclusions

In this work, we present the successful transfer of Au nanoislands formed by the Au evaporation/annealing technique on a solid substrate to a flexible adhesive tape by soft printing approach. This is possible due to specific surface hydrophobization achieved via chemical vapor deposition treatment with perfluosilane. Surface analyses validate that the suggested approach is efficient, convenient, facile, and robust to side-selective AuNI transfer to polymeric materials. Owing to precise control of the AuNI size, soft plasmonic films with unique spectral-selective optical properties were achieved. Since the AuNIs incorporated into polymeric tape remain capping-free, their further modification is possible. The transition of AuNIs from rigid to flexible substrates influences only marginally the plasmonic characteristics of AuNIs mainly due to their partial embedding. It is assumed that not just randomly distributed AuNIs can be soft-printed but also lithographically created metallic arrays with long-range 2D organization. The obtained flexible plasmonic adhesive tapes can be used as optical/tensile/pressure sensors to study the influence of the nanostructured features on soft matter film formation, and *vice versa*; i.e., they may serve as a plasmonic ruler to track the changes in the material at the surficial layer where the AuNIs are sited. Due to the capping-free formation of AuNIs, they can be used for further modification for chemical and biological sensing applications.

## Experimental Section

### Materials

Microscope glass coverslips (Schott AG borosilicate glass D263T No. 3, 24 × 24 mm^2^, with *T*_g_ ≈ 557 °C, ORSAtec, Germany) were used as substrates. Hydrogen peroxide (30%, Bio-Lab Ltd., Israel), sulfuric acid (95–98%, AR-p, Bio-Lab Ltd., Israel), ethanol (Abs, Gadot, Israel), and gold (99.999%, Holland-Moran, Israel or Kurt Lesker, USA) were used as received. Triple distilled water (TDW) was used throughout the experiments (Milli-Q). Transparent or magic opaque Scotch brand tape (3M, USA, base-cellophane; adhesive-a mixture of acrylates) Kapton and Mylar tape were purchased in a local market. Medical scotch tapes (3M, USA): Durapore (base-silk-like cloth), Transpore (base-polyethylene), and Micropore (base-viscose) were purchased in a local medical pharmacy.

### Procedures

#### Glass Substrate Cleaning

Glass coverslips (slides) were cut into 24 × 9 mm sections and cleaned in a glass beaker with a Teflon holder containing freshly prepared “Piranha” solution (H_2_O_2_–H_2_SO_4_, 1:3 by volume) for 1 h (*Caution! The solution is highly aggressive; handle it with care*). Subsequently, the slides were washed 3 times with deionized (DI) and TDW water and finally with absolute ethanol ultrasonically (DU-32, Argo-Lab, Italy) for 10 min each. After the cleaning procedure, a batch of 12 substrates was thoroughly dried under a stream of N_2_.

#### Fabrication of Au Films via Evaporation

The slides were mounted on the metallic holder with metallic clips in the e-beam evaporator. The chamber was evacuated to a pressure of (0.8–4) × 10^–7^ Torr and thin Au films were deposited at a deposition rate of ∼0.1 Å s^–1^, to nominal thicknesses of 1, 3, 5, 7, 9, 11, 13, and 15 nm, *i.e*., the nominal mass thickness as read by the evaporator QCM thickness monitor.

#### Gold Nanoisland (AuNI) Formation

After the evaporation, the slides were annealed at 450 °C for 20 h in a muffle furnace (Ney Vulcan 3–550, Dentsply, USA) in air, at a 3–5 °C min^–1^ heating rate, and then subsequently cooled to room temperature inside a furnace by natural convection. After the fabrication, the slides were stored in a 24-well plastic holder in a desiccator under vacuum (ca. 0.9 bar, in-house vacuum supply). Before use, the slides were kept under such vacuum conditions for ca. 24 h (Figure 1a).

#### Slide Hydrophobization

The slides, with and without AuNIs, were placed in an opened glass Petri dish and put into a desiccator (inner volume is *ca*. 2.6 dm^3^) with a 1.5 mL Eppendorf test tube containing 30 μL of trichloro(1H,1H,2H,2H-perfluorooctyl)silane (97%, Sigma-Aldrich, USA) under constant in-house vacuum supply overnight (*ca*. 12 h, vacuum is *ca*.–0.7 - – 0.9 bar, see Figure S9 and Figure S10). Then, the vacuum valve was opened for 2 h and closed again with the slides inside for an additional 12 h. Of note, the excess volume of the silane solution produces extra HCl, the hydrolysis product that may lead to poor surface hydrophobicity. Furthermore, the excessive hydrochloric acid may react with gold islands resulting in their instability and significant plasmon shifts up to Au layer disintegration as exposed to a liquid. Before the desiccator was opened, the secondary port was connected to a nitrogen supply through a 150 nm filter; N_2_ gas was gently pumped in, rising to ambient pressure with simultaneous vacuum valve closing. Before silanization, the slides might be additionally cleaned with UV/Ozone generator (UVOCS Inc. T10*10/OES/E, USA) for 20 min. In another strategy, 1*H*,1*H*,2*H*,2*H*-perfluorooctyltriethoxysilane (98%, Sigma-Aldrich, USA) can be used instead of fluorinated trichlorosilane. After the silanization process, an adhesive tape was applied on a slide and then unstuck.

### Characterization of Materials

#### UV–Vis Spectroscopy Measurements

Extinction spectra at normal incidence were measured using a Cary 300 Bio (Varian-Agilent, USA) spectrophotometer in a special holder, using air as the baseline. Transmission spectra were recorded in the range of 190–900 nm with a scan rate of 120 nm min^–1^ (average acquisition time per point is 0.5 s).

#### Optical Microscopy Imaging

Optical microscopy images were obtained in dark or bright field modes using a BX-61 (Olympus, Japan) microscope equipped with Leica Flexcam C3 digital camera and ×5 to ×100 objectives (UMPlanFI and MPlanFL N).

#### Field Emission High-Resolution Scanning Electron Microscopy (FE-HRSEM)

High-resolution scanning electron microscopy images were obtained using a Carl Zeiss Ultra-55 and SIGMA Ultrahigh-resolution SEM with an accelerating voltage of 3 kV under a vacuum of <5 × 10^–5^ mbar and a working distance of ∼4 mm. The slides or films were placed on Al stubs and fixed with carbon tape (2SPI, USA). The samples were then partially coated with carbon paste (2SPI, USA), and 2–3 nm of iridium (Safematic CCU-010 HV high vacuum sputter coater, LabTech, U.K.) was deposited immediately before imaging to improve sample conductivity and image contrast.

#### Energy Dispersive X-ray Spectrometry Analysis (XEDS)

Energy dispersive X-ray spectrometry analysis was performed using a SIGMA Ultrahigh-resolution SEM (Carl Zeiss, Germany) with an accelerating voltage of 2–20 kV using a XFlash 6130 QUANTAX EDS detector (Bruker, USA) under a vacuum of <5 × 10^–5^ mbar and a working distance of ∼6.5 mm. The EDS analysis was performed on the same samples that were used for FE-HRSEM imaging. Several samples were used without any additional coating to exclude signal interference.

#### AuNI Size Distribution Statistical Analysis

Top-view FE-HRSEM images were used for statistical analysis of the AuNIs diameter using the ImageJ 1.6 software. First, the known distance in pixels was converted to the distance in nanometers. Then, a threshold was applied to identify the area of AuNIs. In the case of low contrast, the enhanced contrast filter was applied with a 0.35% value of saturated pixels. AuNIs diameter was calculated using the Analyze particles mode excluding NPs on edges. An elliptical model was applied to fit the AuNIs shape. 500 AuNIs were used for every deposited nominal mass thickness to calculate the major/minor diameter, surface coverage, and aspect ratio.

#### Contact Angle (CA) Measurements

The water contact angle (WCA) measurements were performed by a contact angle goniometer (Theta Flex, Biolin Scientific, China, Germany) at room temperature. The drop volume for the measurements was ∼6 μL, and the drop profile was captured on camera. The drop profile was fitted by using the OneAttension analysis software provided by the manufacturer. The WCA was calculated for the sessile drop shape by using the Laplace–Young fitting method. The contact angle values specified in the text are averaged by at least three independent measurements.

#### Atomic Force Microscopy (AFM)

The samples were imaged using an AFM JPK Nano wizard 4 (Germany) and assembled with an Olympus optical microscope (Japan) for sample finding using AC160 or AC240 cantilevers in a tapping mode. The images were processed with JPK data processing software and Gwiddion 1.6.

#### Numerical Simulations of AuNIs Embedding

A COMSOL Multiphysics 5.6 software was used for all-optical simulations based on a finite element method. The AuNIs were modeled as a truncated spheroid with a flat top surface of 23 and 130 nm in diameter and a height of 25 nm, embedded 5, 50, and 95% into the 500 nm-thick substrate slab. The surrounding medium was air. Rectangular-packed AuNIs with a center-to-center distance of 150 and 300 nm were used in the calculations of 23 and 130 nm in diameter, respectively, to eliminate evanescent electromagnetic field coupling between NPs. Periodic and port boundary conditions were used in the horizontal and vertical directions, respectively. Constant refractive indices of 1.0 and 1.5 were used for air and substrate, respectively. The excitation light was linearly polarized and incident near-normally from the airside of the sample. For Au, dispersive dielectric constants from the literature were used.^58^

#### Raman Spectroscopy Measurements

Raman scattering spectra and mapping measurements in the range from 800 to 2000 cm^–1^ were collected from the sample films using the backscattering mode. A LabRAM HR Evolution confocal micro-Raman spectrometer (Horiba, France) equipped with four lasers and laser power control was used for the measurements. For the experiments, the 633 nm laser was used with a spot size of 78.5 μm^2^ (using the macrospot mode with a circular spot having a diameter of ∼10 μm) to average over sample inhomogeneity and reduce the power on the sample thereby reducing some of the fluorescence, and the field enhancement of SERS which can result in photochemical effects. The LabRAM is fitted with an 800 mm spectrograph with high spectral resolution and low stray light. Frequency calibration was performed before every measurement session on the characteristic Si Raman peak at 520.7 cm^–1^ by using a single-crystal Si wafer. The measurements were recorded with a 600 grooves mm^–1^ grating with ∼1.3 cm^–1^ pixel resolution. The samples were illuminated using a microscope x50 objective (LMPlanFL N, numerical aperture (NA) = 0.5, Olympus, Japan). The system utilizes a confocal modular microscope (Olympus BX-FM) with a spatial resolution better than 1 μm for the 633 nm laser. The Raman spectra were collected using a 1024 × 256 pixels open electrode front-illuminated CCD camera (Syncerity, Horiba, USA), which was cooled to −60 °C. The spectra were baseline-corrected using a polynomial as is commonly done. The spectral collection was from the sticky side of scotch tape, onto which an aqueous solution of 0.1 mM Rhodamine 6G (>95%, Sigma-Aldrich, USA) was drop-cast, left for 10 min in a closed Petri dish with a wet piece of paper to maintain constant humidity, and then gently rinsed with Milli-Q water and dried.

## Supporting Information Available

Gold nanoislands processing scheme, unannealed gold films characterization, FE-HRSEM imaging and statistical distribution analysis of AuNIs on glass substrates before silanization, AFM imaging of AuNIs on glass substrates before silanization, slide hydrophobization process, FE-HRSEM imaging and statistical distribution analysis on AuNIs on glass substrates after silanization, FE-HRSEM imaging and statistical distribution analysis on AuNIs on polymeric substrates, AuNIs transfer onto other tape forms, completeness of AuNIs transfer, application of 1*H*,1*H*,2*H*,2*H*-perfluorooctyltriethoxysilane, multiplasmonic systems, patterning applications, AuNIs transfer onto medical/surgical tapes, AuNIs transfer into other polymers, COMSOL simulations of AuNIs embedding, and contact angle measurements and surface hydrophobicity (PDF)
Soft-print process of gold nanoislands on scotch adhesive tape (MP4)
