# Supplementary material for: Flexible Soft-Printed Polymer Films with Tunable Plasmonic Properties
Source: ACS Mater Au. 2023 Sep 5;3(6):699–710. doi: 10.1021/acsmaterialsau.3c00023 (PMC10636776; doi:10.1021/acsmaterialsau.3c00023)
Supplement: Supplementary file 1 — mg3c00023_si_001.pdf [file mg3c00023_si_001.pdf]

SUPPLEMENTARY INFORMATION

FLEXIBLE SOFT-PRINTED POLYMER FILMS WITH TUNABLE PLASMONIC PROPERTIES

Aleksei Solomonov[1], Anna Kozell[1], Alexander B. Tesler[2], Iddo Pinkas[3], Seth Walensky[1], and Ulyana Shimanovich[1]*

[1]Department of Molecular Chemistry and Materials Science, Weizmann Institute of Science, Rehovot 7610001, Israel.

[2]Department of Materials Science and Engineering, Friedrich-Alexander-Universität Erlangen-Nürnberg, Martensstrasse 7, Erlangen 91056, Germany.

[3]Chemical Research Support, Weizmann Institute of Science, Rehovot 7610001, Israel.

Contents

1. Gold Nanoislands processing scheme 1
2. Unannealed gold films characterization 3
3. FE-HRSEM imaging and statistical distribution analysis of AuNIs on glass substrates before silanization 4
4. AFM imaging of AuNIs on glass substrates before silanization 7
5. Slide hydrophobization process 8
6. FE-HRSEM imaging and statistical distribution analysis on AuNIs on glass substrates after silanization 9
7. FE-HRSEM imaging and statistical distribution analysis on AuNIs on polymeric substrates 12
8. AuNIs transfer onto other tape forms 14
9. Completeness of AuNIs transfer 16
10. Application of 1H,1H,2H,2H-Perfluorooctyltriethoxysilane 18
11. Multiplasmonic systems 19
12. Patterning applications 20
13. AuNIs transfer onto medical/surgical tapes 23
14. AuNIs transfer into other polymers 33
15. COMSOL simulations of AuNIs embedding 34
16. Contact angle measurements and surface hydrophobicity 35

1. Gold Nanoislands processing scheme

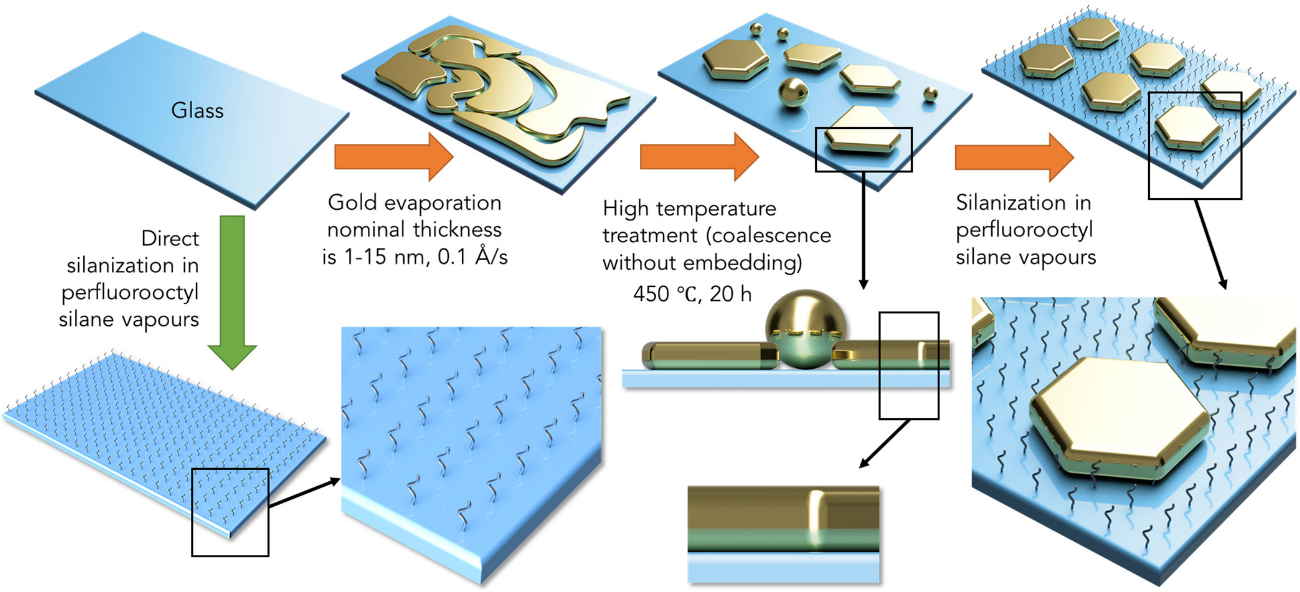

Figure S1. *Scheme of AuNIs formation on glass and slides silanization. Images were generated with Autodesk Fusion 360.*

2. Unannealed gold films characterization

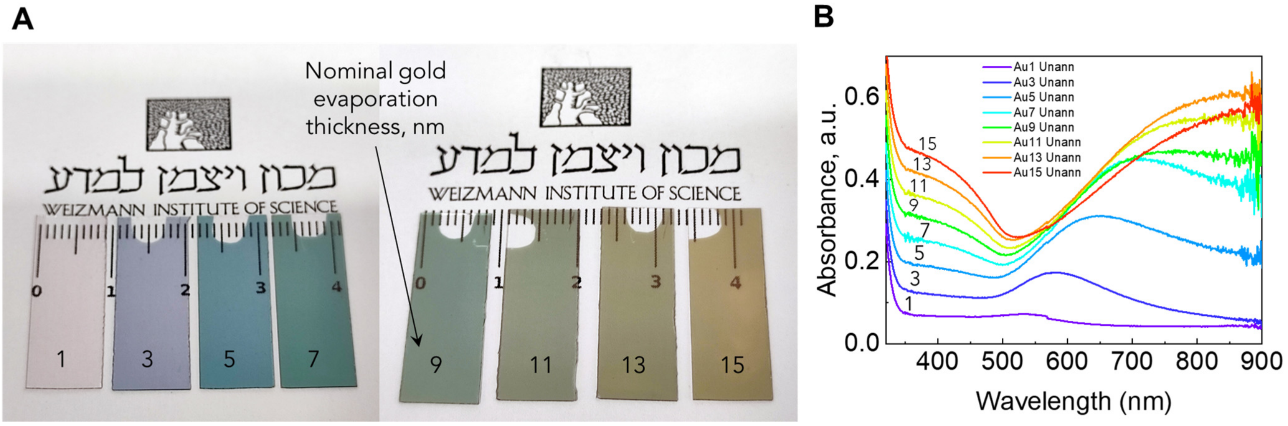

Figure S2. (***a***) *Digital images of glass slides with gold after evaporation, images courtesy of the Weizmann Institute of Science (the logo was printed and the slides were places on the paper with it also demonstrating the optical properties of the slides with respect to their transparency), the ruler is in cm and* (***b***) *the corresponding UV-vis spectra of the slides.*

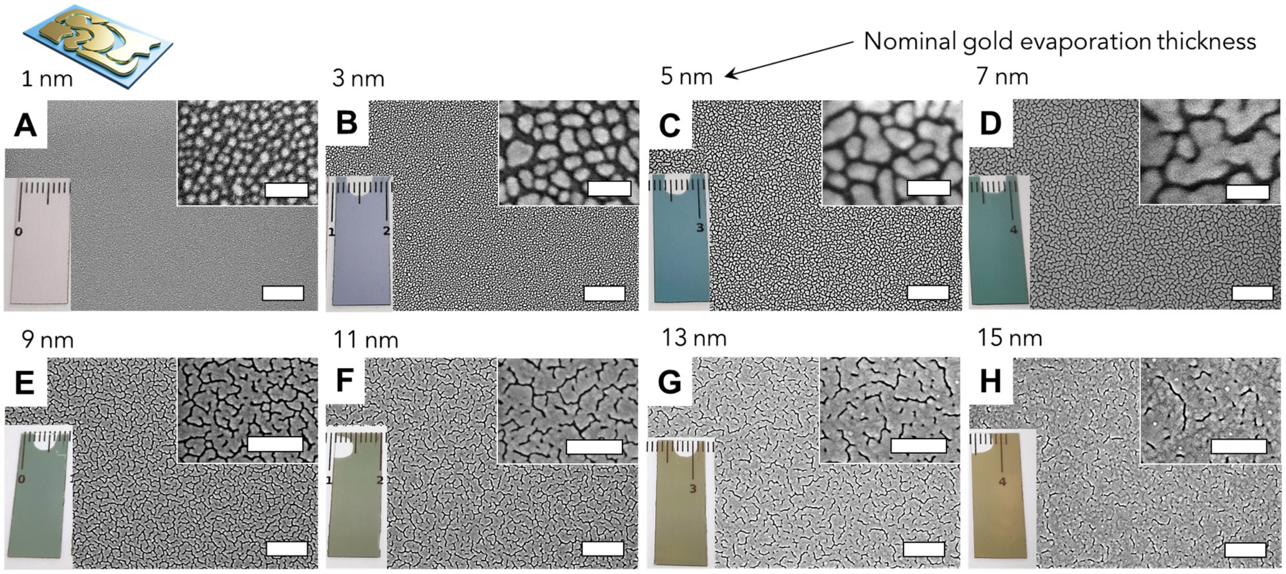

Figure S3. *FE-HRSEM images of glass slides with gold after evaporation of gold (gold nominal thickness is:* ***a*** *– 1 nm,* ***b*** *– 3 nm,* ***c*** *– 5 nm,* ***d*** *– 7 nm,* ***e*** *– 9 nm,* ***f*** *– 11 nm,* ***g*** *– 13 nm, and* ***h*** *– 15 nm), insets – detailed images of the nanostructured gold surface and corresponding digital images of the slides in bottom left corner (referred to the Figure S2). Scalebars for all the images are 300 nm (the scalebar of insets of* ***a–d*** *is 40 nm, the scalebar of insets of* ***e–h*** *is 200 nm); all the FE-HRSEM images were taken from the in-lens detector.*

3. FE-HRSEM imaging and statistical distribution analysis of AuNIs on glass substrates before silanization

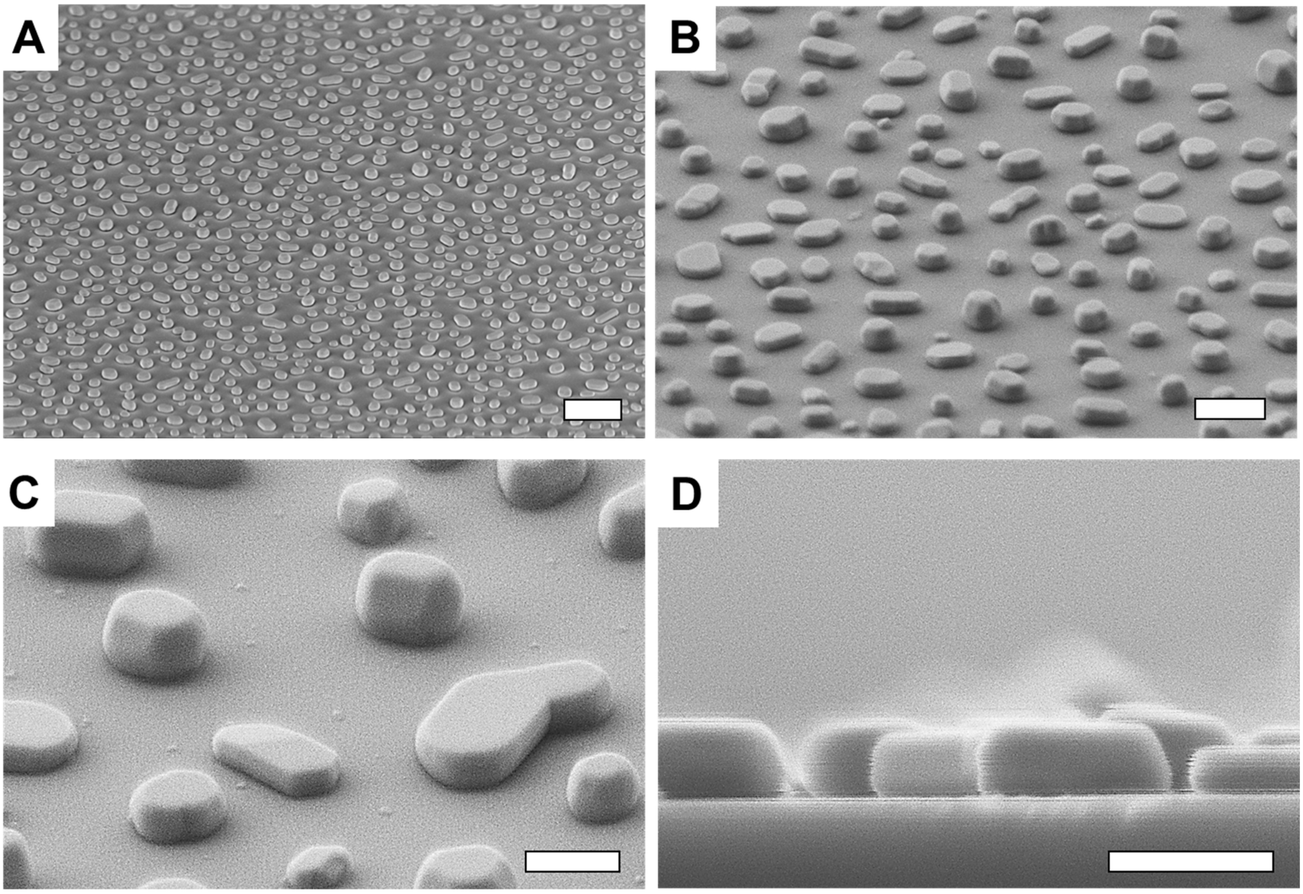

Figure S4. *FE-HRSEM of AuNIs formed by gold film of (15 nm nominal thickness) annealed at 450 ℃ at different magnifications and different angles of imaging. The scalebar for* ***(a)*** *is 1 µm,* ***(b)*** *– 400 nm and* ***(c, d)*** *– 200 nm* ***(b, d)****, images of* ***(b-d)*** *were taken from the SE2 detector, whereas the image of* ***(a)*** *was from the in-lens detector.*

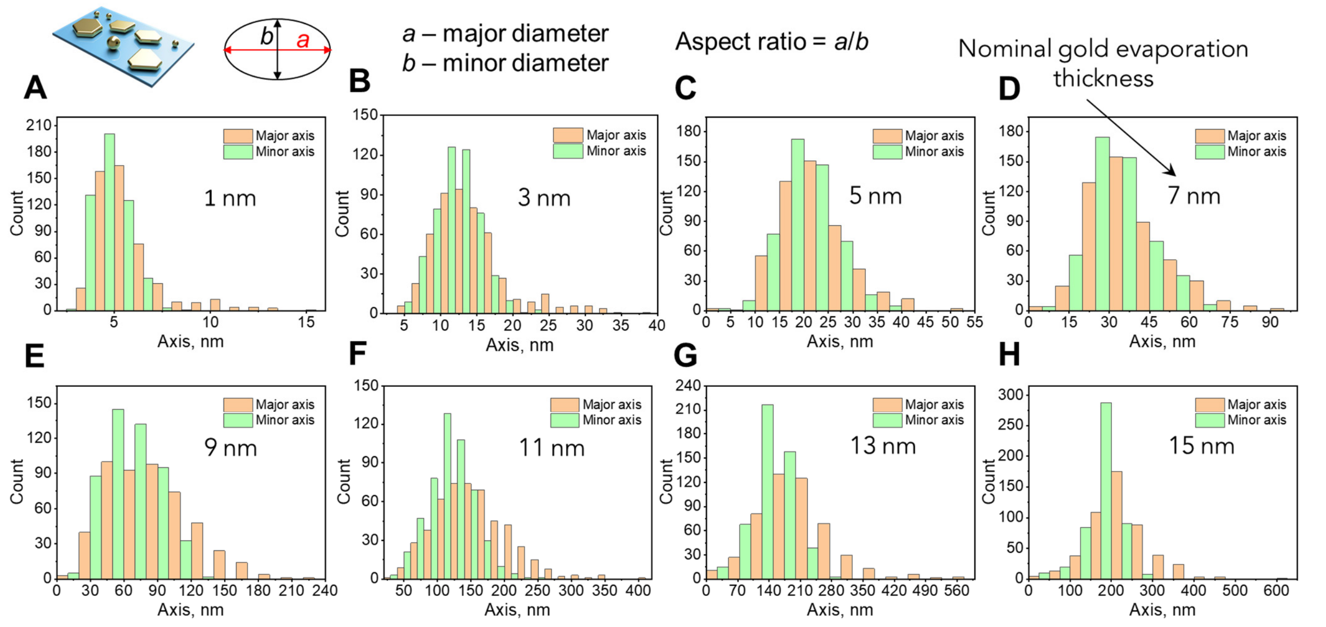

Figure S5. *Distribution statistics of AuNIs on glass (nominal gold evaporation thickness:* ***a*** *– 1 nm,* ***b*** *– 3 nm,* ***c*** *– 5 nm,* ***d*** *– 7 nm,* ***e*** *– 9 nm,* ***f*** *– 11 nm,* ***g*** *– 13 nm, and* ***h*** *– 15 nm).*

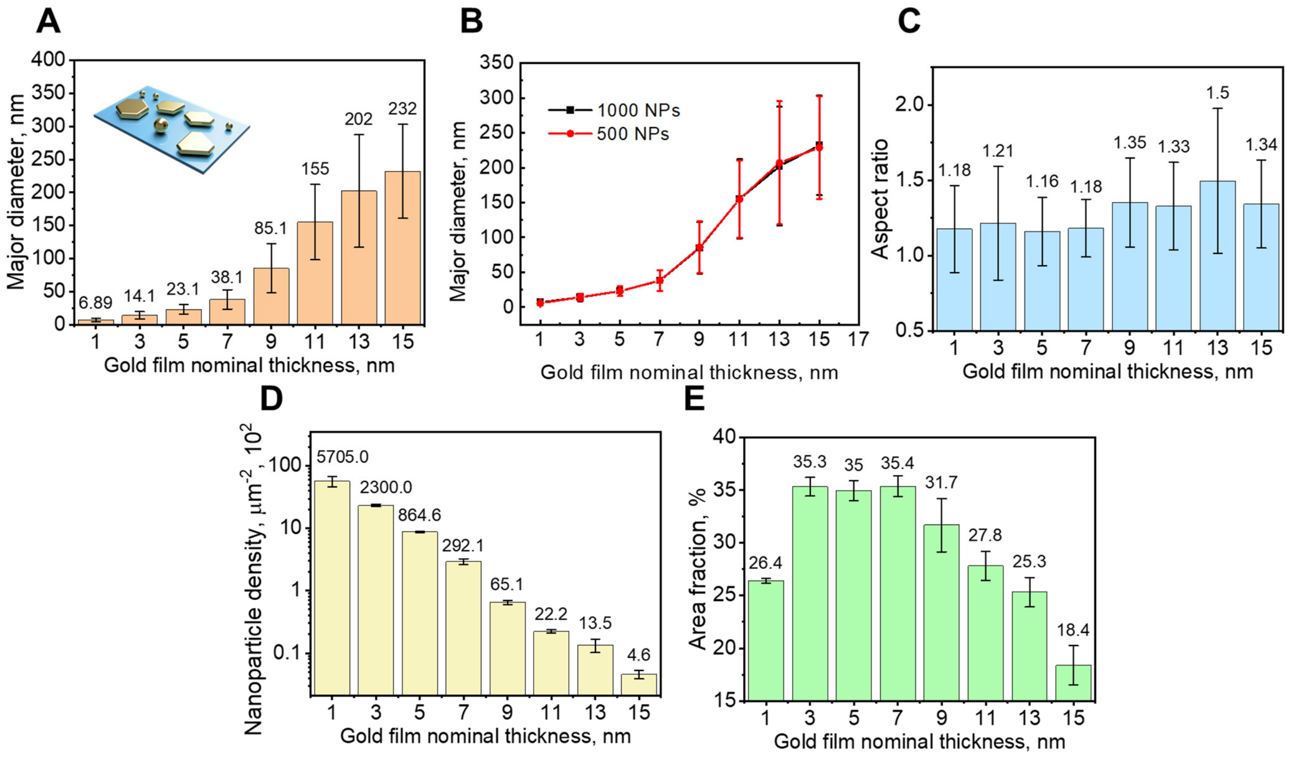

Figure S6. *Summary of the distribution statistics of AuNIs on glass: gold nominal evaporation thickness vs. (****a****) AuNI major diameter, (****b****) Comparison of the AuNI major diameter vs. the gold nominal evaporation thickness at two values of nanoparticles selected for sampling, and (****c****) the aspect ratio, (****d****) the AuNI density on the surface unit, and (****e****) the surface coverage of AuNIs on the slide.*

All AuNIs of type I are preferentially spherical, with an aspect ratio of 1.16 – 1.21, whereas the AuNIs of type II show an aspect ratio in the range of 1.33 – 1.5 (**Figure S6c**) possessing pita-like shape. Note that in this range, splitting of the LSPR band into longitudinal and transversal sections is not expected.

4. AFM imaging of AuNIs on glass substrates before silanization

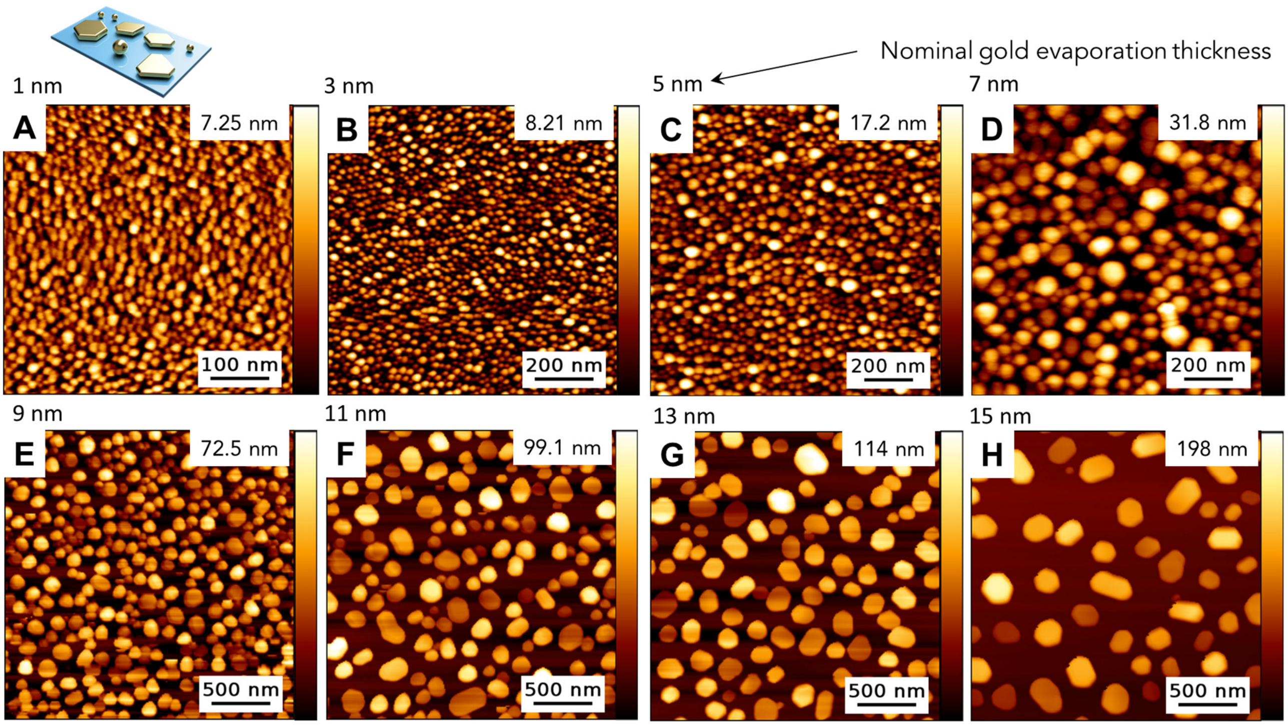

Figure S7. *AFM images of AuNIs on glass (nominal gold evaporation thickness:* ***a*** *– 1 nm,* ***b*** *– 3 nm,* ***c*** *– 5 nm,* ***d*** *– 7 nm,* ***e*** *– 9 nm,* ***f*** *– 11 nm,* ***g*** *– 13 nm, and* ***h*** *– 15 nm).*

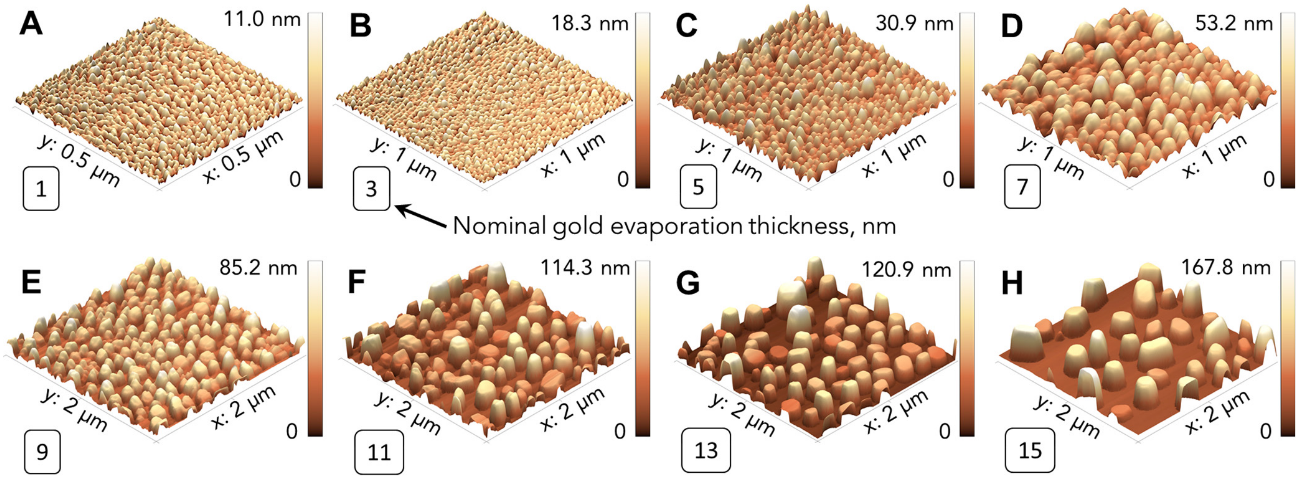

Figure S8. *3D AFM images of AuNIs on glass (nominal gold evaporation thickness:* ***a*** *– 1 nm,* ***b*** *– 3 nm,* ***c*** *– 5 nm,* ***d*** *– 7 nm,* ***e*** *– 9 nm,* ***f*** *– 11 nm,* ***g*** *– 13 nm, and* ***h*** *– 15 nm).*

5. Slide hydrophobization process

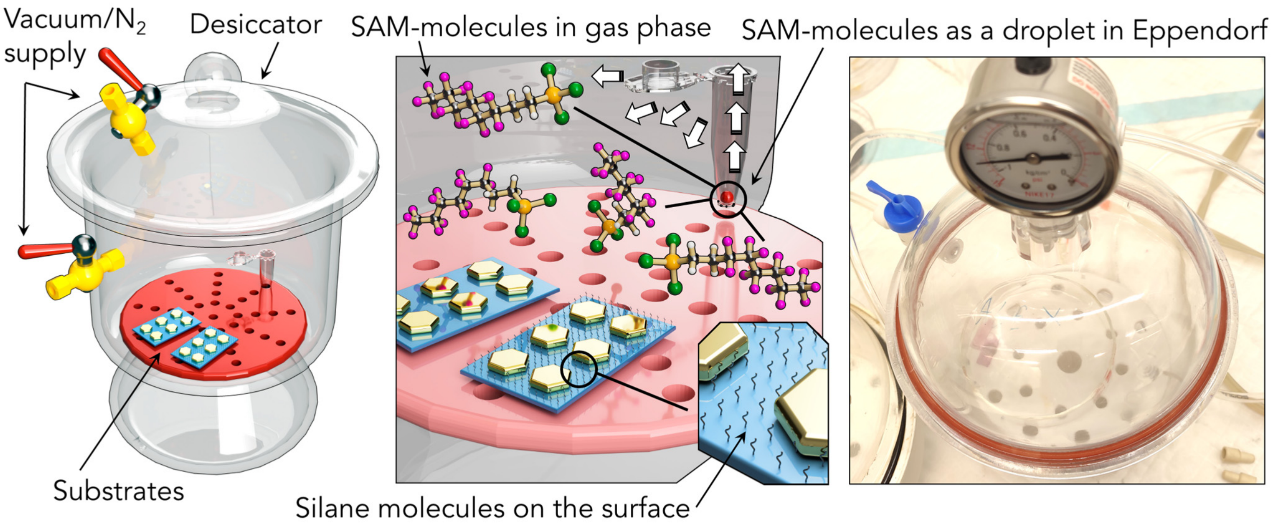

Figure S9. *Schematic illustration of the substrates' silanization process and a digital image of the desiccator with the samples inside (right). Left and middle images were modelled in Autodesk Fusion 360.*

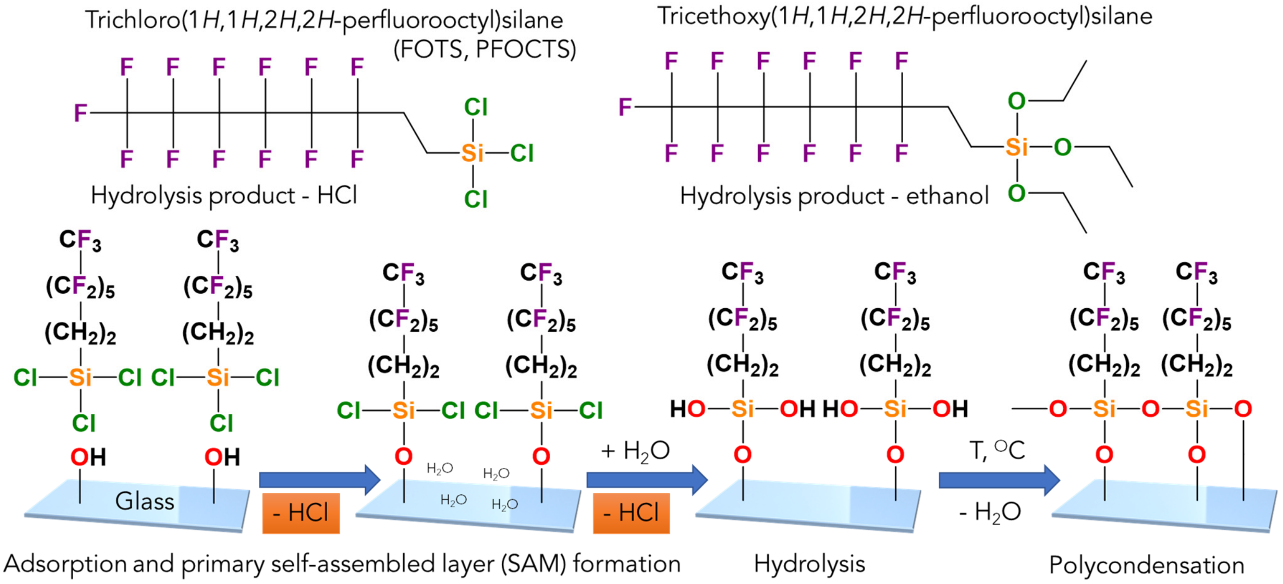

Figure S10. *Structural formulas of Trichloro(1H,1H,2H,2H-perfluorooctyl)silane (FOTS, PFOCTS) and triethoxy(1H,1H,2H,2H-perfluorooctyl)silane and a scheme of the silanization process using the example of FOTS.*

6. FE-HRSEM imaging and statistical distribution analysis on AuNIs on glass substrates after silanization

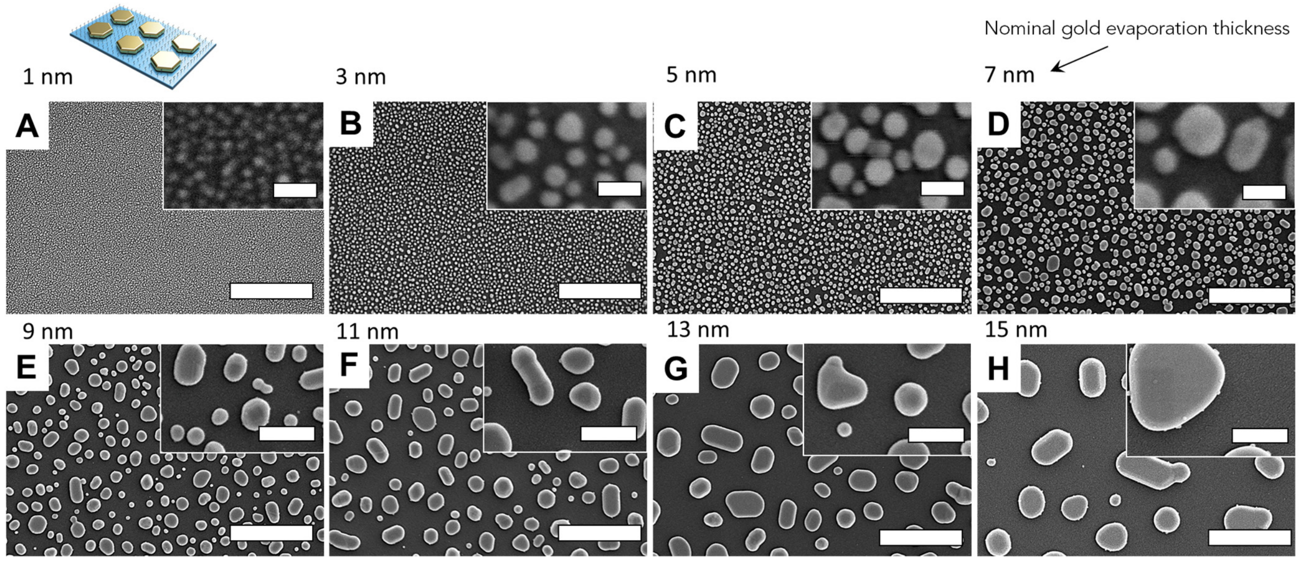

Figure S11. *FE-HRSEM images of AuNIs on glass after silanization (nominal gold evaporation thickness: **a** – 1 nm, **b** – 3 nm, **c** – 5 nm, **d** – 7 nm, **e** – 9 nm, **f** – 11 nm, **g** – 13 nm, and **h** – 15 nm). The scalebar for (**a-h**) is 600 nm, for insets of (**a-d**) it is 40 nm, and for (**e-h**) it is 200 nm; all the images were taken from an in-lens detector.*

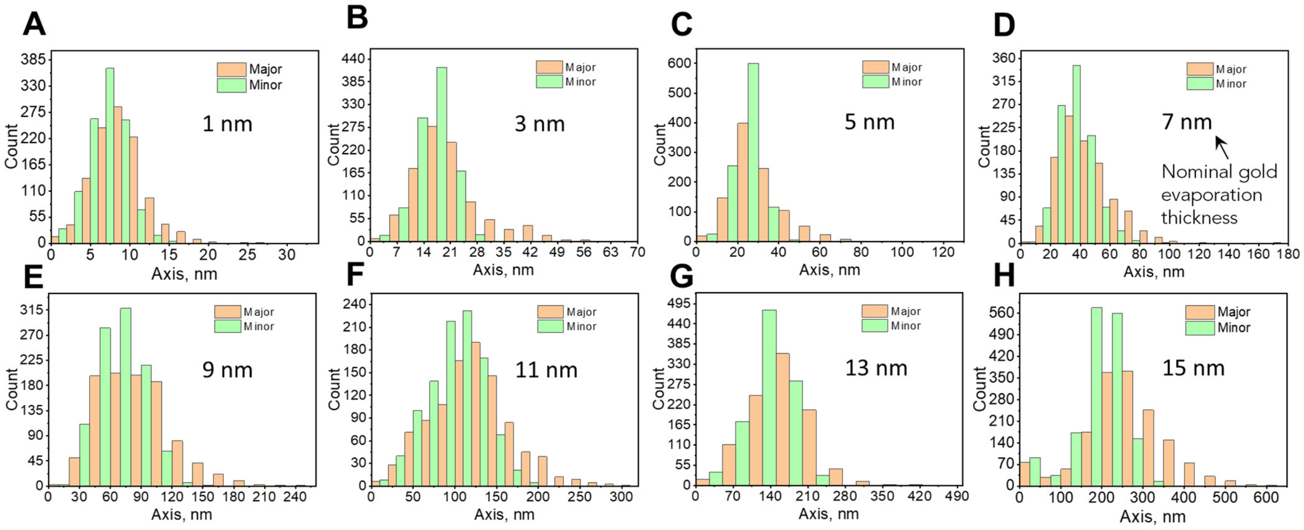

Figure S12. *Distribution statistics of AuNIs on glass after silanization (nominal gold evaporation thickness: **a** – 1 nm, **b** – 3 nm, **c** – 5 nm, **d** – 7 nm, **e** – 9 nm, **f** – 11 nm, **g** – 13 nm, and **h** – 15 nm).*

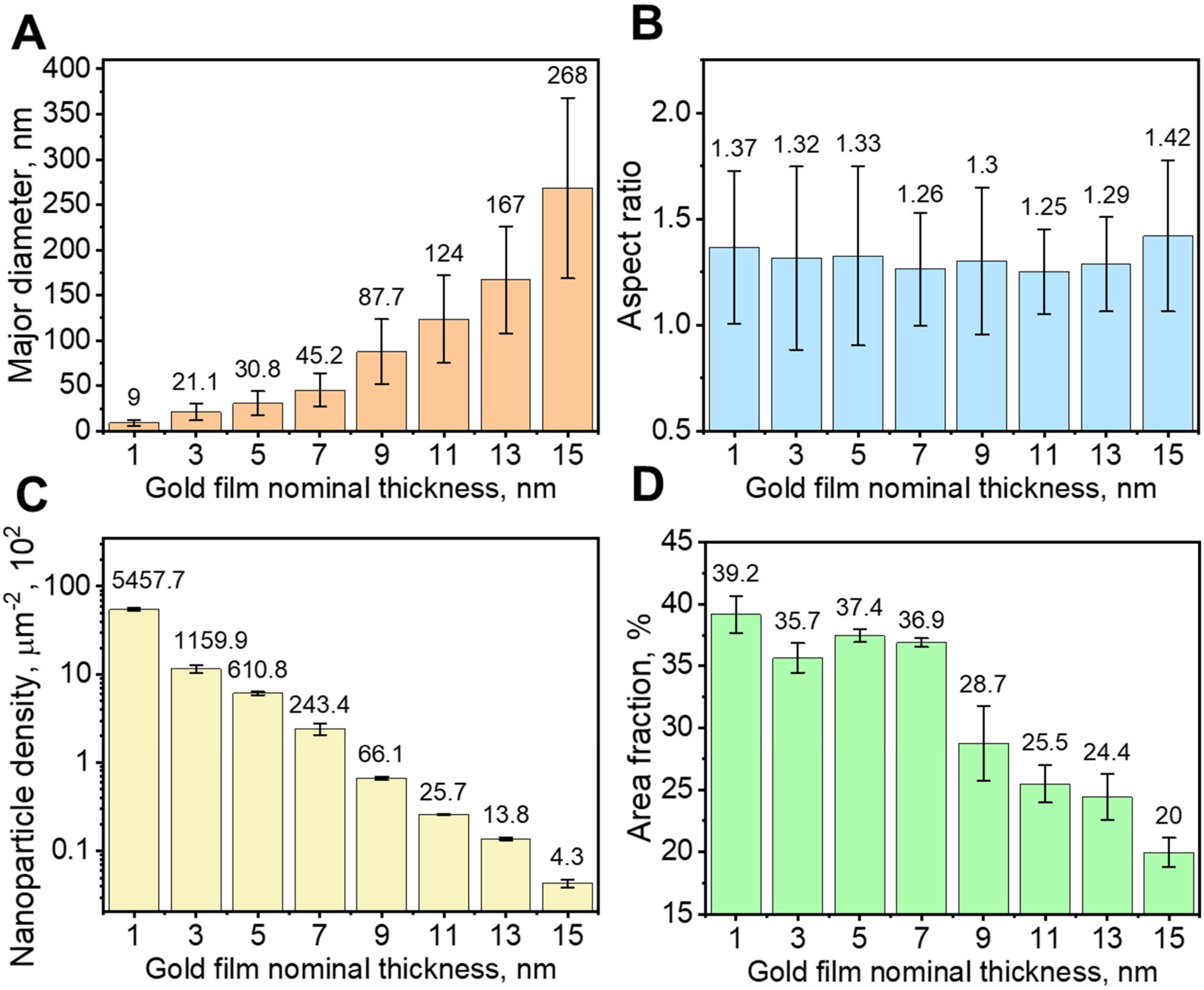

Figure S13. *Summary of the distribution statistics of AuNIs on glass after silanization: gold nominal evaporation thickness vs. (**a**) the AuNI major diameter, (**b**) the aspect ratio, (**c**) the AuNI density on the surface unit, (**d**) the surface coverage of AuNIs on the slide.*

To achieve maximal transfer of AuNIs to the soft substrates, the following conditions should be fulfilled. (*1*) A priori, the evaporated thin Au films on glass slides should undergo solid-state dewetting\coalescence to form well-defined crystalline NIs during high-temperature annealing. (*2*) The high-temperature treatment should be optimized to the specific temperatures, *i.e.*, preferentially below the glass transition temperature of the substrate to prevent embedding and stabilization of AuNIs; otherwise, the soft-printing process would be significantly hindered and even impossible since partially-embedded AuNIs are stable enough to be resistant to solvent treatments.[1,2]

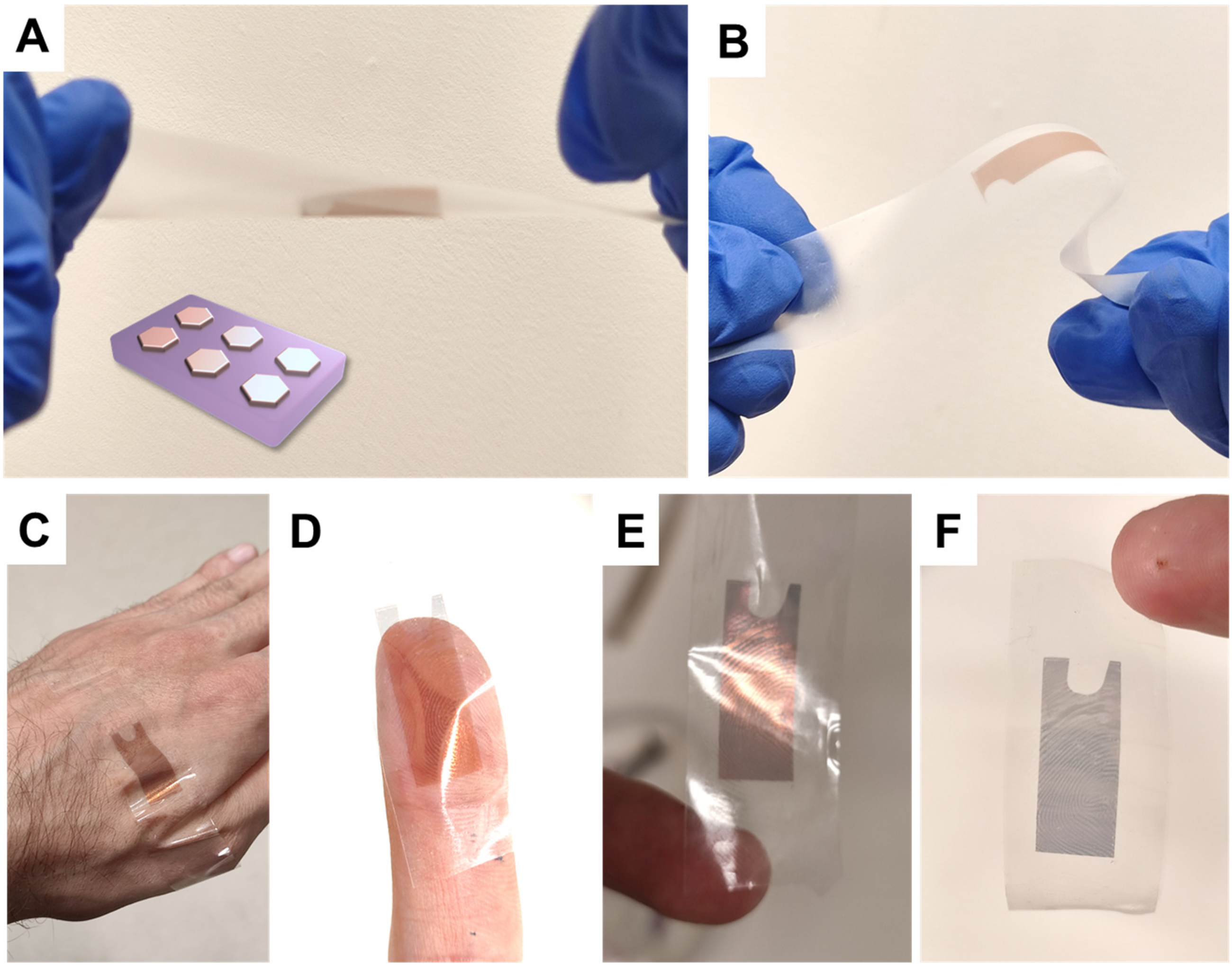

Figure S14. *Basic mechanical properties of the AuNIs-scotch tape films: (**a**) twisting and (**b**) bending. Applying the tape on the skin: (**c**) a way to stick the films on the skin, (**d**) scotch tape with embedded AuNIs stuck to a finger and (**e, f**) a fingerprint pattern appeared on the tape after its removal.*

7. FE-HRSEM imaging and statistical distribution analysis on AuNIs on polymeric substrates

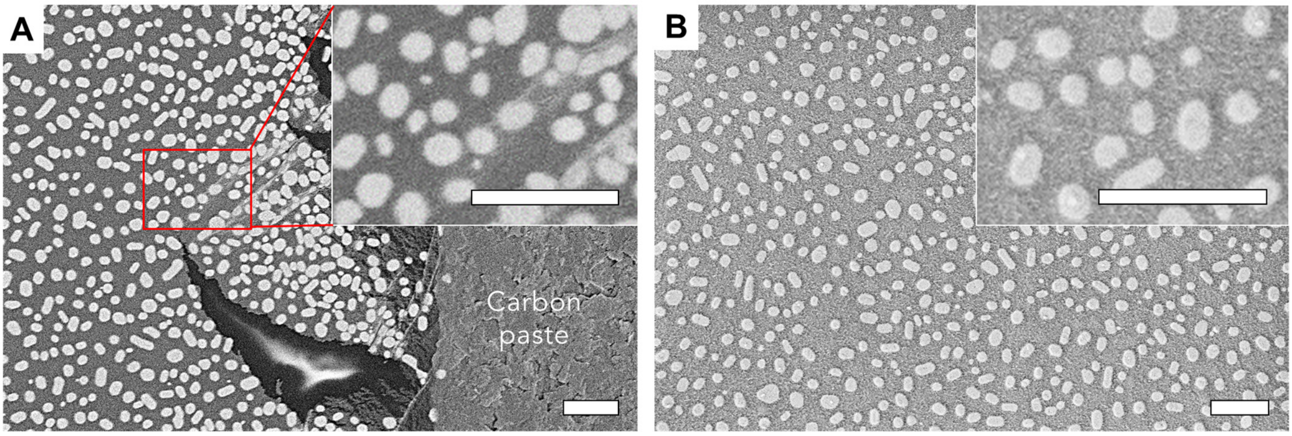

Figure S15. *FE-HRSEM images of the scotch tape with AuNIs (the nominal gold evaporation thickness is 15 nm) taken from (**a**) an ESB detector and (**b**) an SE2 detector. Insets – magnified images. The scalebar for all the images is 1 µm.*

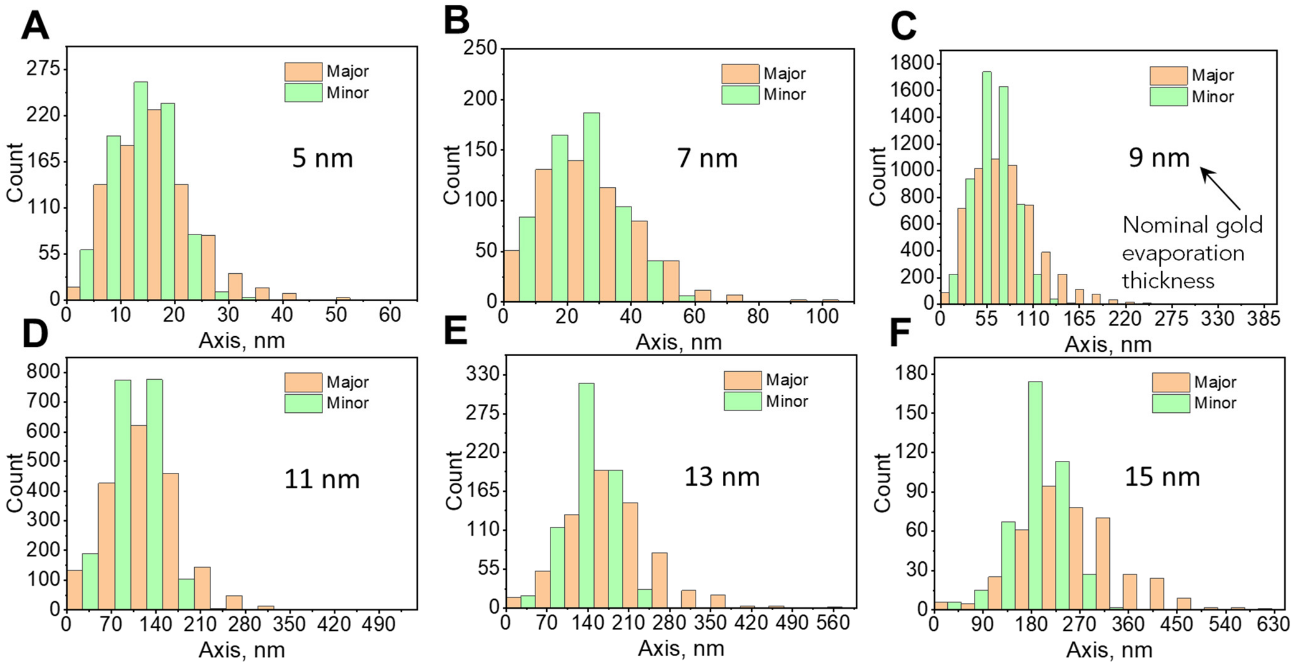

Figure S16. *Distribution statistics of AuNIs after transferring onto a scotch tape (nominal gold evaporation thickness: **a** – 5 nm, **b** – 7 nm, **c** – 9 nm, **d** – 11 nm, **e** – 13 nm, and **f** – 15 nm. Statistical distribution analysis of AuNIs of 1 and 3 nm of gold evaporation thickness was unable to be complete due to insufficient contrast of the corresponding images).*

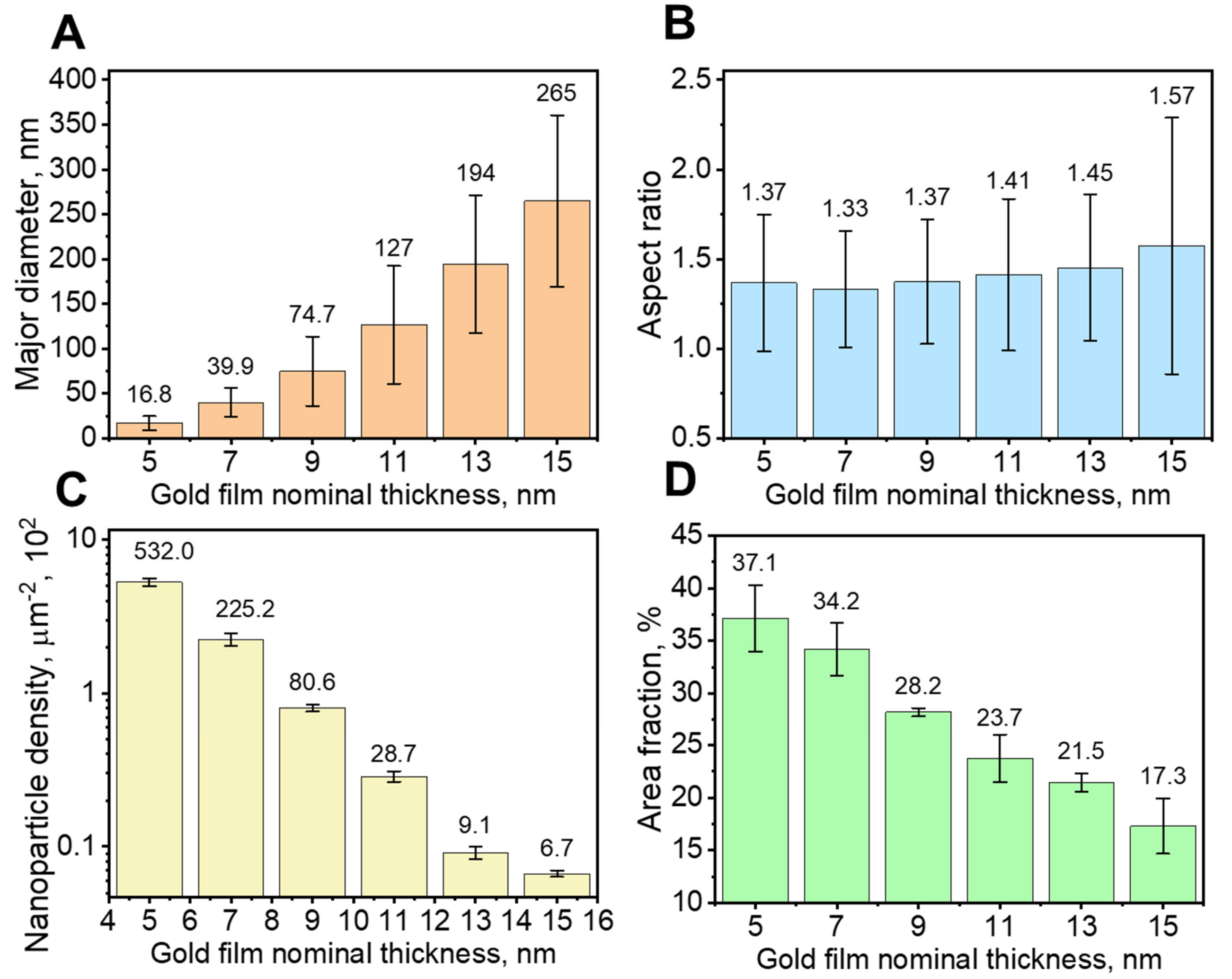

***Figure S17**. Summary of the distribution statistics of AuNIs after transferring onto a scotch tape: gold nominal evaporation thickness vs. (**a**) AuNI major diameter, (**b**) the aspect ratio, (**c**) the AuNI density on the surface unit, (**d**) the surface coverage of AuNIs on the slide.*

8. AuNIs transfer onto other tape forms

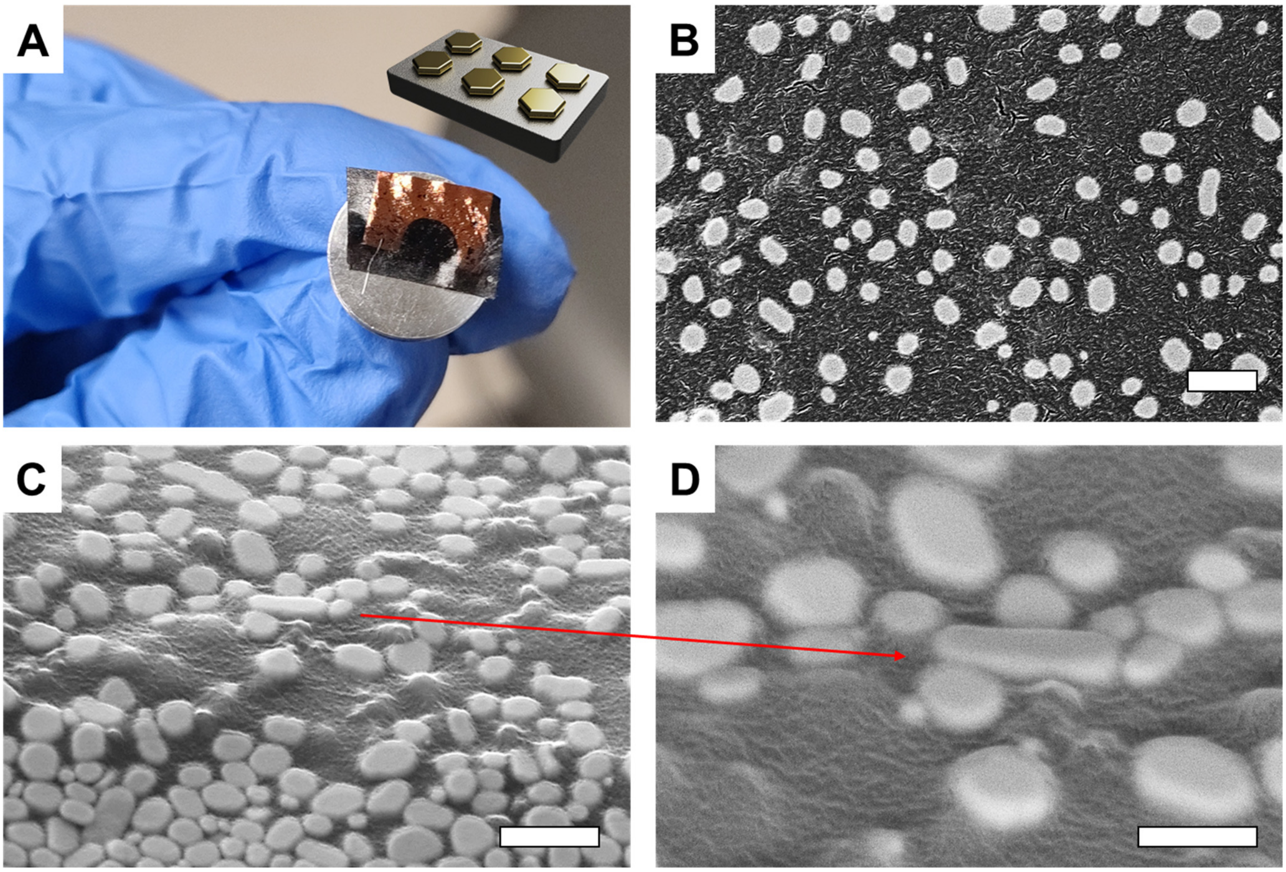

***Figure S18**. (**a**) Digital image of a carbon tape piece, attached to an SEM stub, with embedded AuNIs of 15 nm of nominal gold evaporation thickness. (**b-d**) FE-HRSEM of the carbon tape with embedded AuNIs taken at different magnifications. The scalebar for (**b, c**) is 400 nm, for (**d**) it is 200 nm. Images of (**b**) and (**d**) were taken from an in-lens detector, while the image of (**c**) was taken from SE2 detector. Arrow shows the specific area on the image taken with high magnification.*

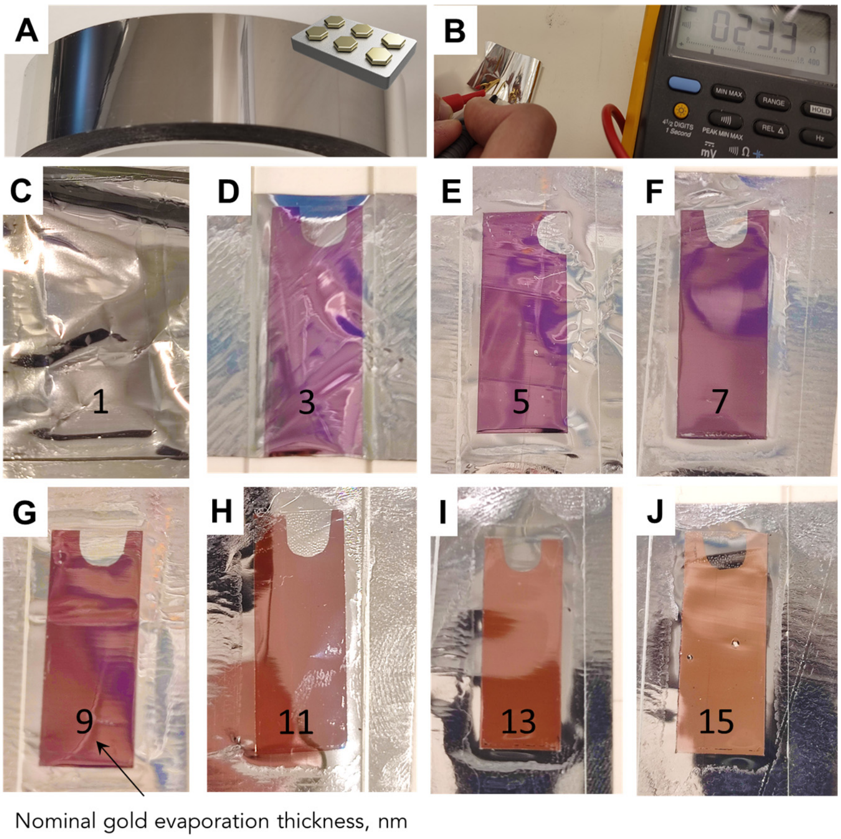

Figure S19. *Digital images of **(a)** reflective metalized tape, **(b)** its conductive properties, and **(c-j)** the same tape after AuNIs were transferred from the glass substrate (nominal gold evaporation thickness: **c** – 1 nm, **d** – 3 nm, **e** – 5 nm, **f** – 7 nm, **g** – 9 nm, **h** – 11 nm, **i** – 13 nm, and **j** – 15 nm).*

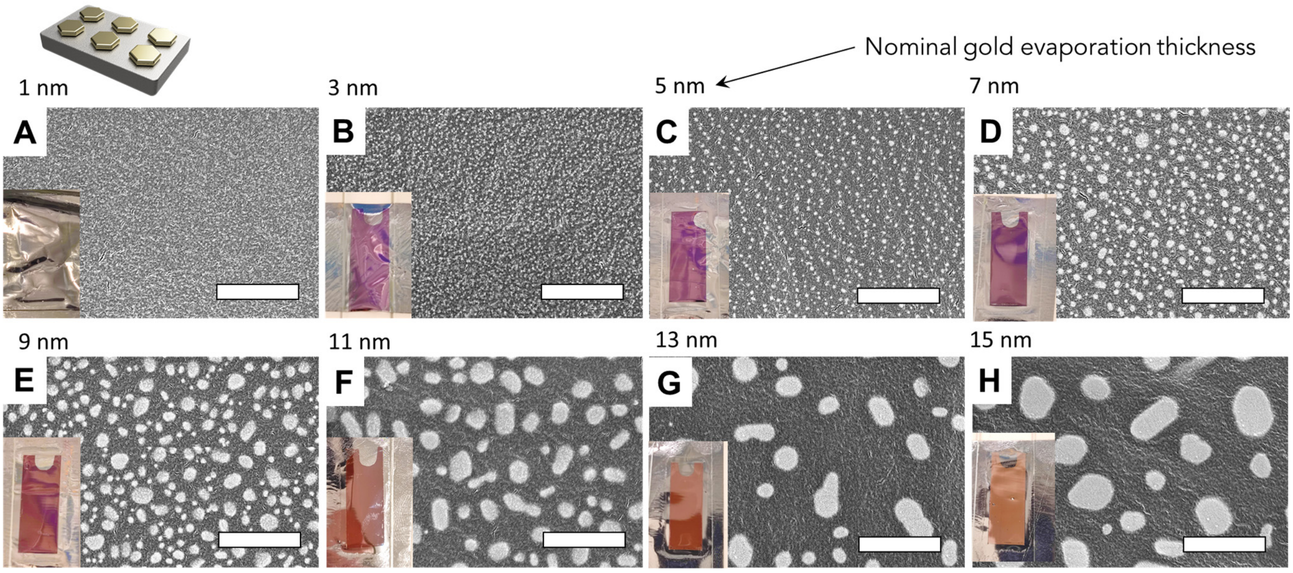

Figure S20. *(**a**) FE-HRSEM of reflective metalized mylar tape after AuNI transfer from the glass substrate (nominal gold evaporation thickness: **b** – 1 nm, **c** – 3 nm, **d** – 5 nm, **e** – 7 nm, **f** – 9 nm, **g** – 11 nm, **h** – 13 nm, and **i** – 15 nm). Insets – digital images of the tape with AuNIs (referred to the Figure S19) The scalebar for all the images is 600 nm; images were taken from an in-lens detector.*

9. **Completeness of AuNIs transfer**

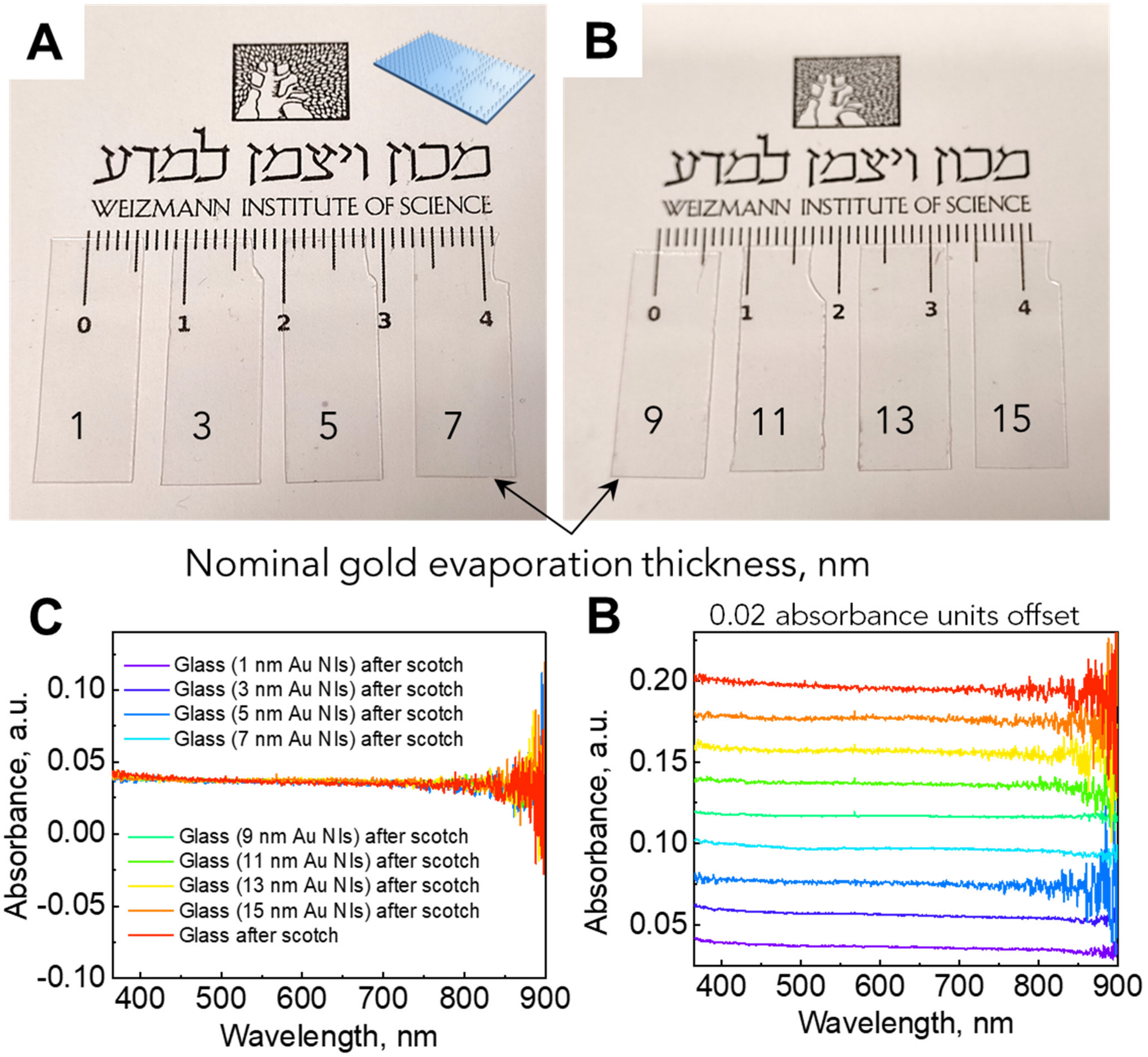

Figure S21. *Digital images of* ***(a-d)*** *the slides after peeling off the scotch tape* ***(a, b)*** *and* ***(c, d)*** *the corresponding UV-vis spectra of the slides after peeling off the scotch tape* ***(c)****: overlaid and* ***(d)*** *separated by an offset of 0.02 a.u. Images courtesy of the Weizmann Institute of Science.*

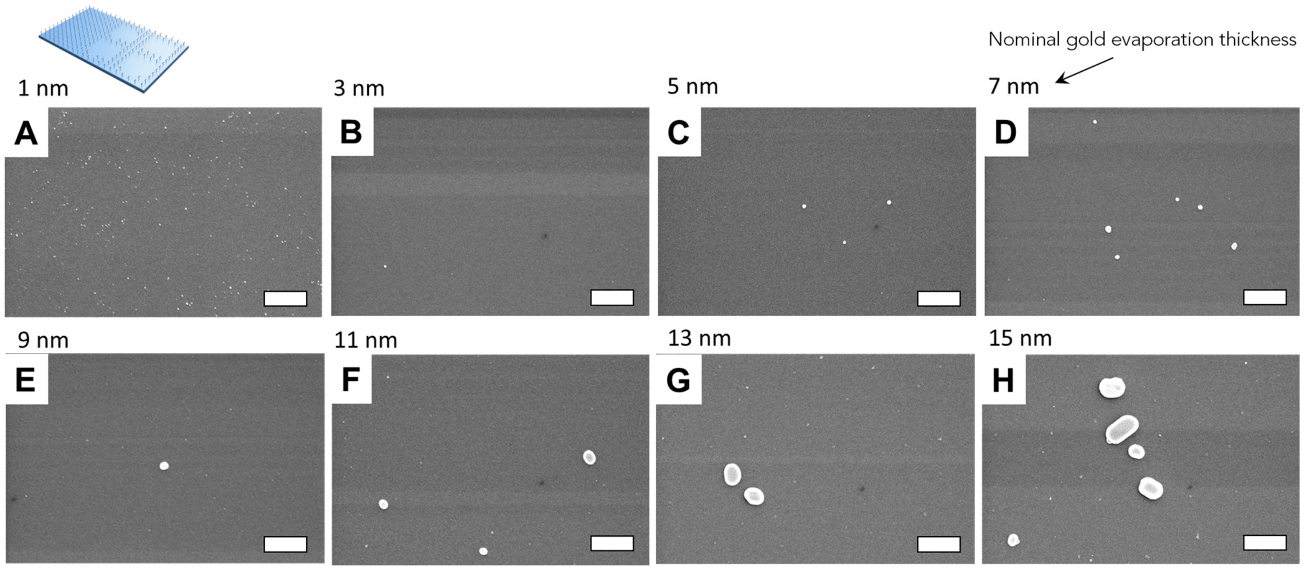

Figure S22. *FE-HRSEM of glass slides after applying scotch tape and AuNI incorporation onto these films, demonstrating the completeness of the AuNI transfer process (gold evaporation thickness):* ***a*** *– 1 nm,* ***b*** *– 3 nm,* ***c*** *– 5 nm,* ***d*** *– 7 nm,* ***e*** *– 9 nm,* ***f*** *– 11 nm,* ***g*** *– 13 nm, and* ***h*** *– 15 nm. The scalebar for all the images is 300 nm. Images were taken from an in-lens detector.*

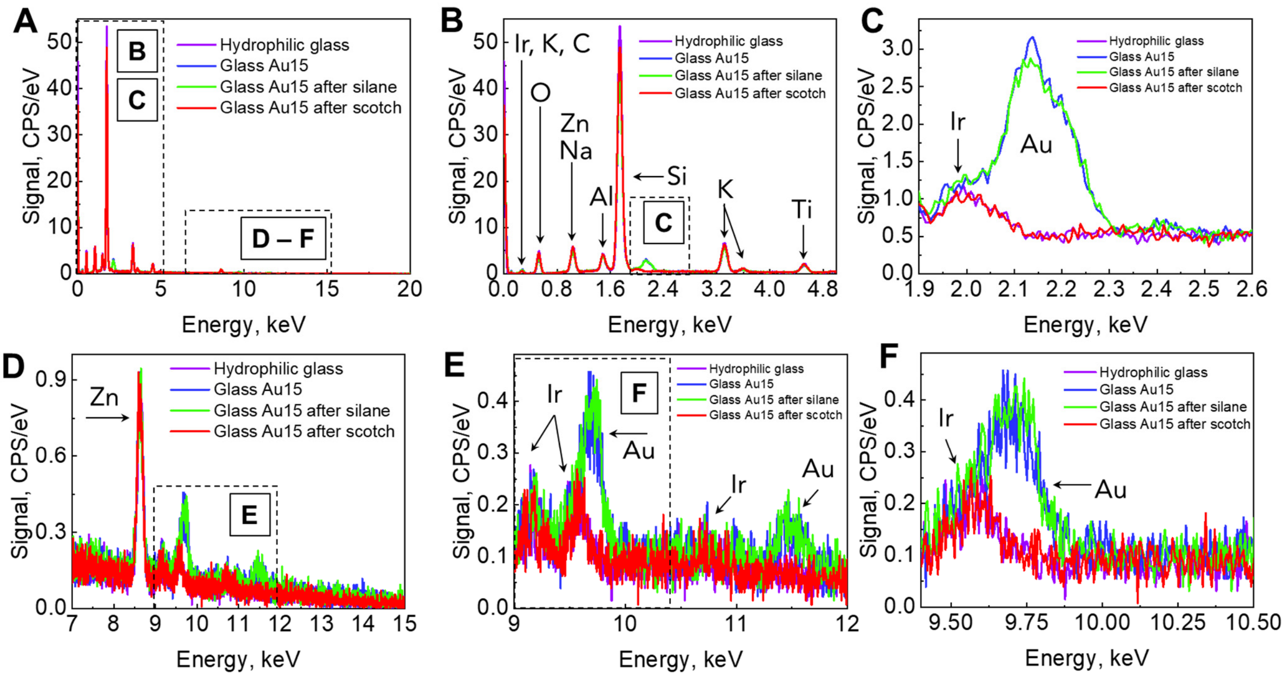

Figure S23. *(**a-f**) EDX spectra of the glass slides covered with 2.5 nm of iridium after cleaning with Piranha (hydrophilic glass), glass slides with AuNIs (the gold evaporation thickness is 15 nm) before and after hydrophobization, and after applying the scotch tape followed by its removal.*

10. Application of 1H,1H,2H,2H-Perfluorooctyltriethoxysilane

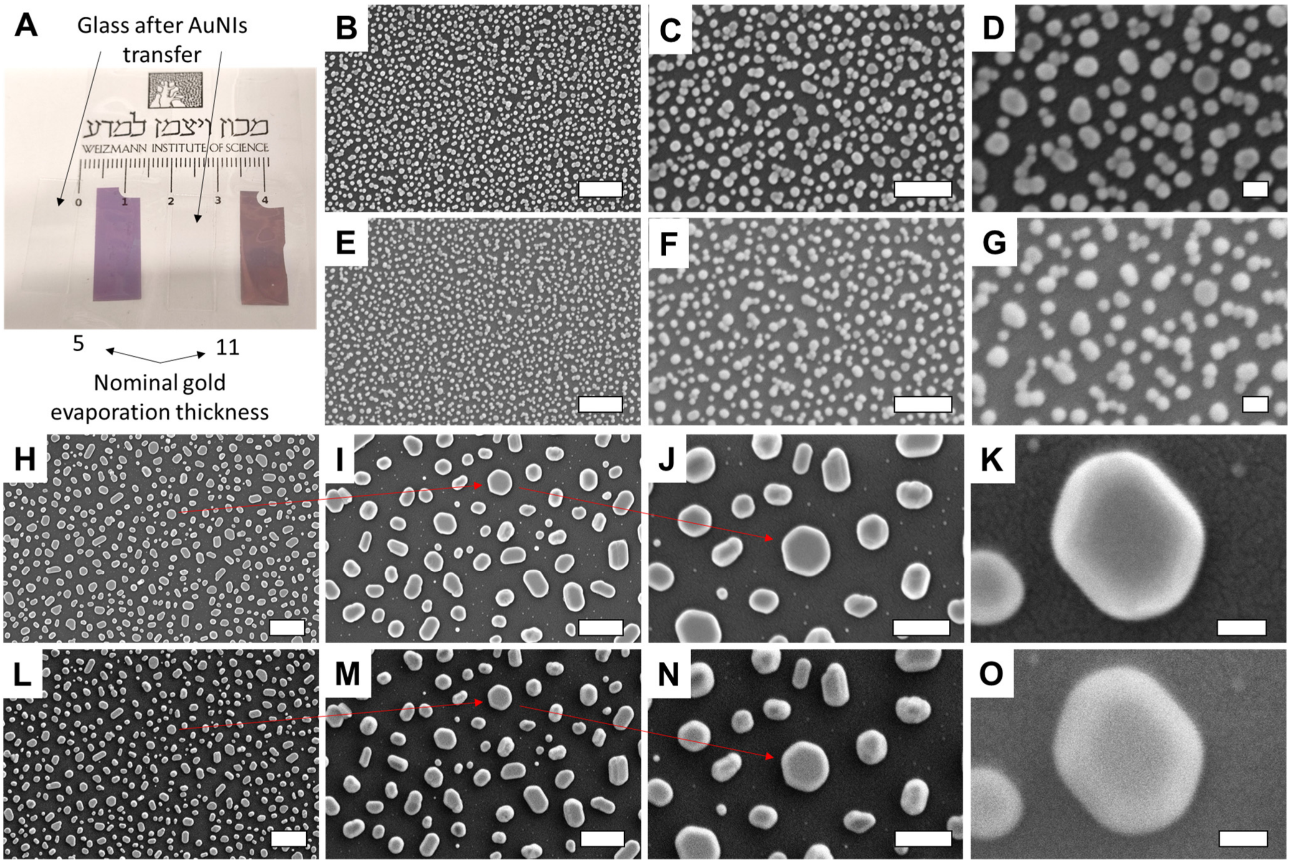

Figure S24. ***(a)*** *Digital images of the scotch tape with transferred AuNIs (5 and 11 nm of nominal gold evaporation thickness) as well as glass slides after AuNIs transfer. Images courtesy of the Weizmann Institute of Science.* ***(b)*** *FE-HRSEM images of glass slides with AuNIs taken at different magnifications (5 and 11 nm of nominal gold evaporation thickness) after pre-treatment with UV-ozone of 30 min and hydrophobization with perfluorooctyltriethoxysilzne. Scalebar for* ***(b, e, i, m)*** *is 300 nm,* ***(c, f, j, n)*** *– 200 µm,* ***(d, g, k, o)*** *– 40 nm,* ***(h, l)*** *– 600 nm. Images of* ***(b-d*** *and* ***h-k)*** *were taken form in-lens detector while images of* ***(e-g*** *and* ***l-o)*** *were taken form SE2 detector. Arrows show the specific area on the images taken with high magnification.*

11. Multiplasmonic systems

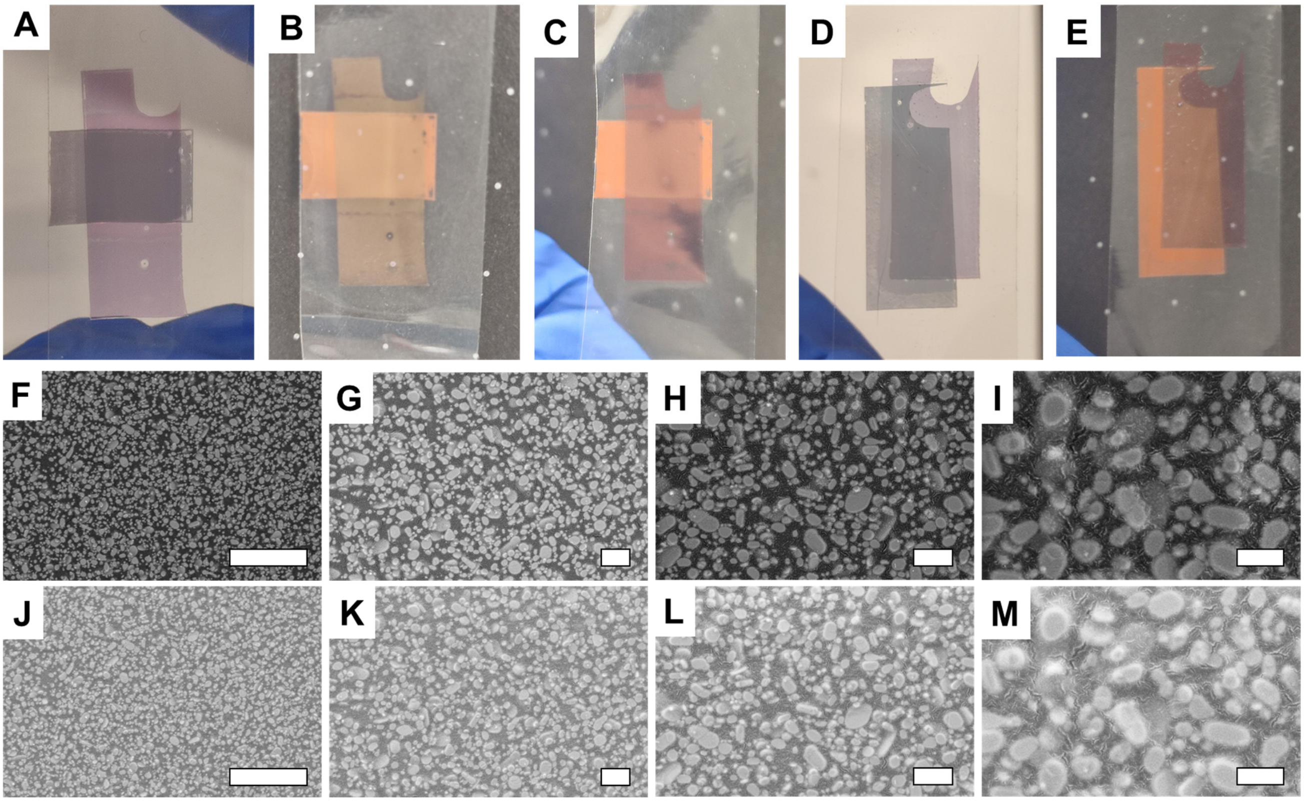

Figure S25. *(**a-e**) Digital images of the scotch tape with sequentially transferred AuNIs of 5 and 11 nm (nominal gold evaporation thickness) at different illumination and transfer modes. FE-HRSEM images of scotch tape. Scalebars for (**f, j**) is 2 µm, (**g, h, k, l**) – 400 nm, and (**i, m**) – 200 nm. Images of (**f-i**) were taken from an in-lens detector and (**j-m**) – from an SE2 detector.*

12. Patterning applications

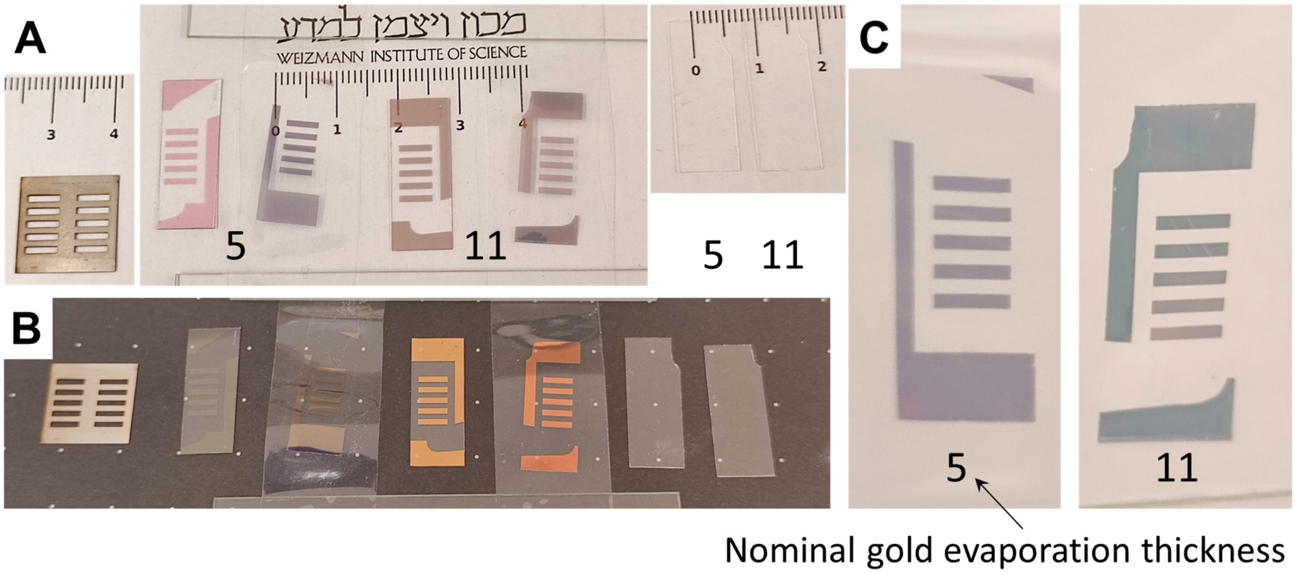

Figure S26. *Digital images of the scotch tape with transferred AuNIs at (**a**) a reflectance mode on a white background, image courtesy of the Weizmann Institute of Science, (**b**) on a black background, and (**c**) in transmittance mode on a white background. Description of the element on the images of (**a** and **b**), from left to right: a shadow evaporation mask, a glass slide of AuNIs of 5 nm of nominal evaporation thickness, the corresponding scotch tape, a glass slide of AuNIs of 11 nm of nominal evaporation thickness, the corresponding scotch tape, glass slides that contained AuNI patterns of 5 and 11 nm of nominal gold evaporation thickness after peeling off the scotch tape.*

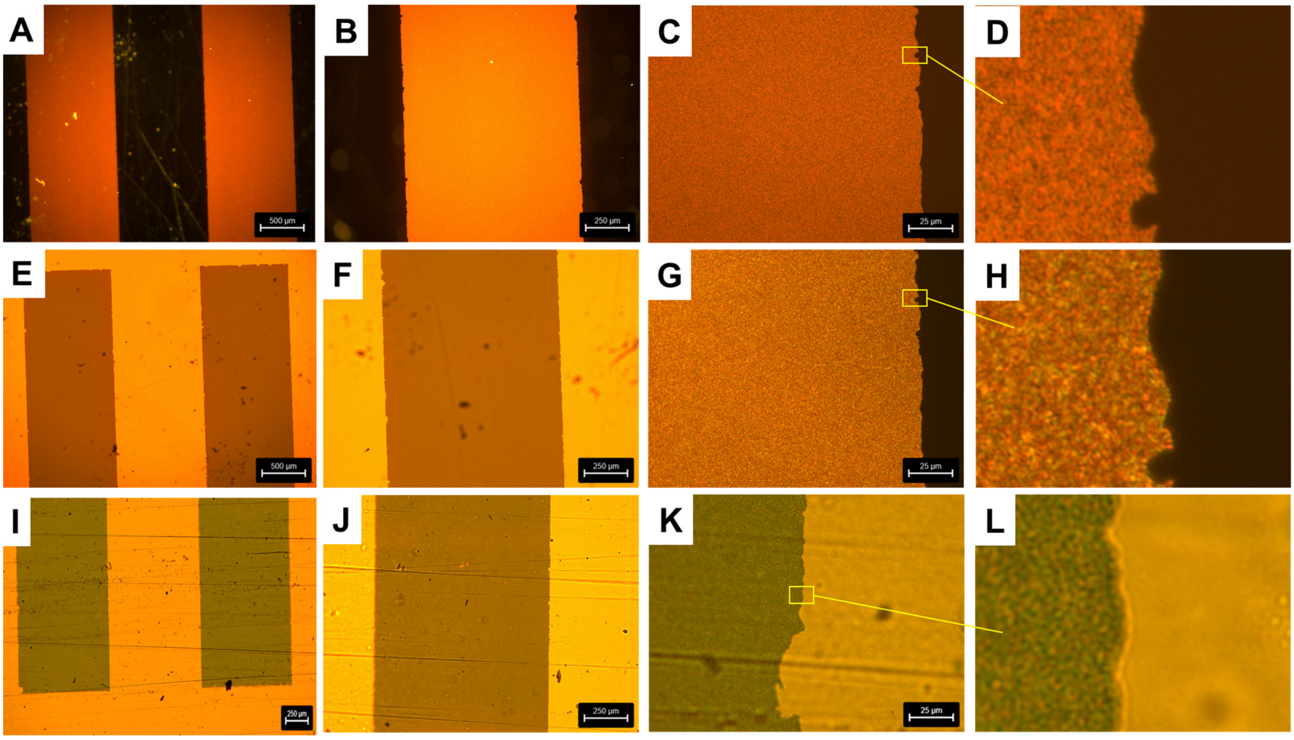

Figure S27. *Optical microscopy images at different scales and different illumination modes of glass slides with an AuNI (11 nm of nominal gold evaporation thickness) pattern:* ***(a-d)*** *– dark field mode,* ***(e-h)*** *– reflectance bright field mode and* ***(i-l)*** *– transmittance mode.*

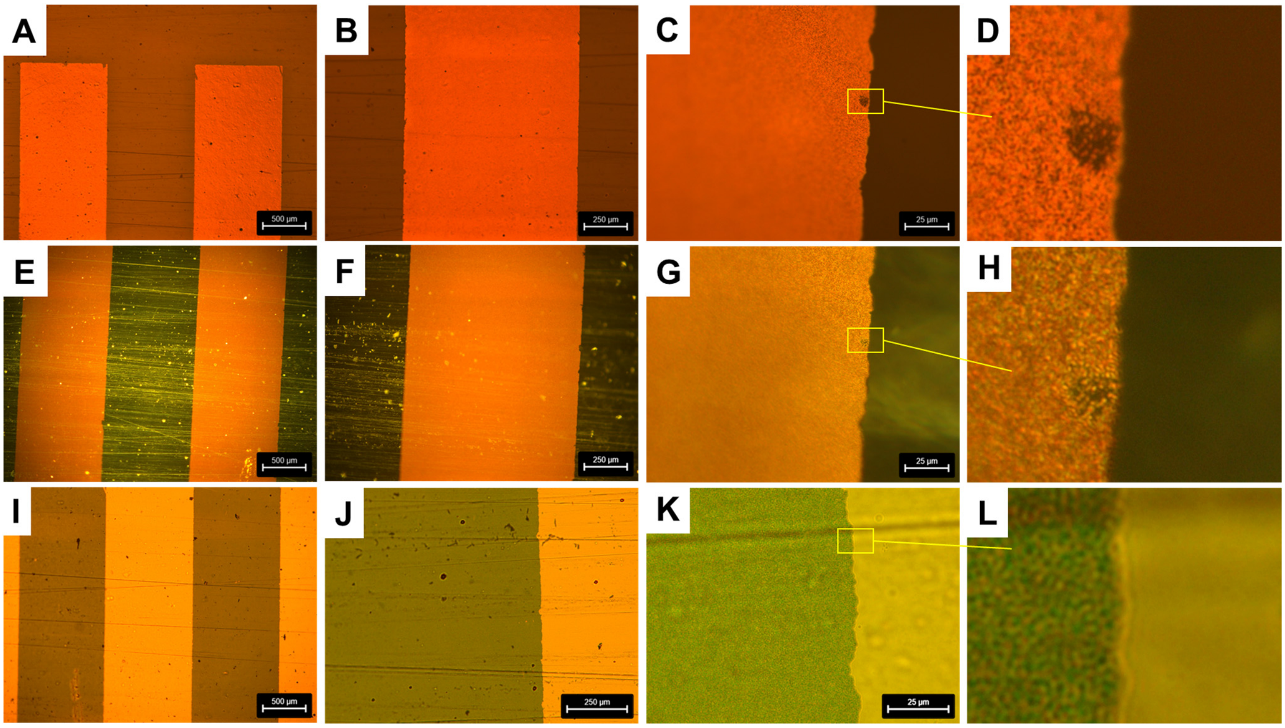

Figure S28. *Optical microscopy images at different scales and different illumination modes of scotch tape with a transferred AuNI (11 nm of nominal gold evaporation thickness) pattern:* ***(a-d)*** *– dark field mode,* ***(e-h)*** *– reflectance bright field mode and* ***(i-l)*** *– transmittance mode.*

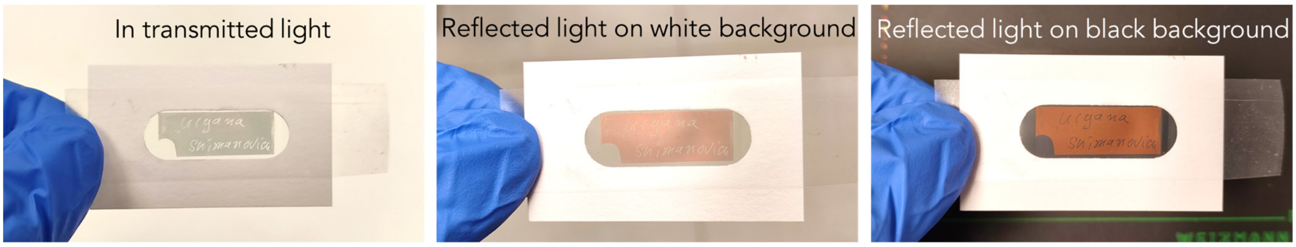

Figure S29. *Digital images of the scotch tape with transferred AuNIs (the nominal gold evaporation thickness is 13 nm) with a manually drawn label in different illumination regimes.*

13. AuNIs transfer onto medical/surgical tapes

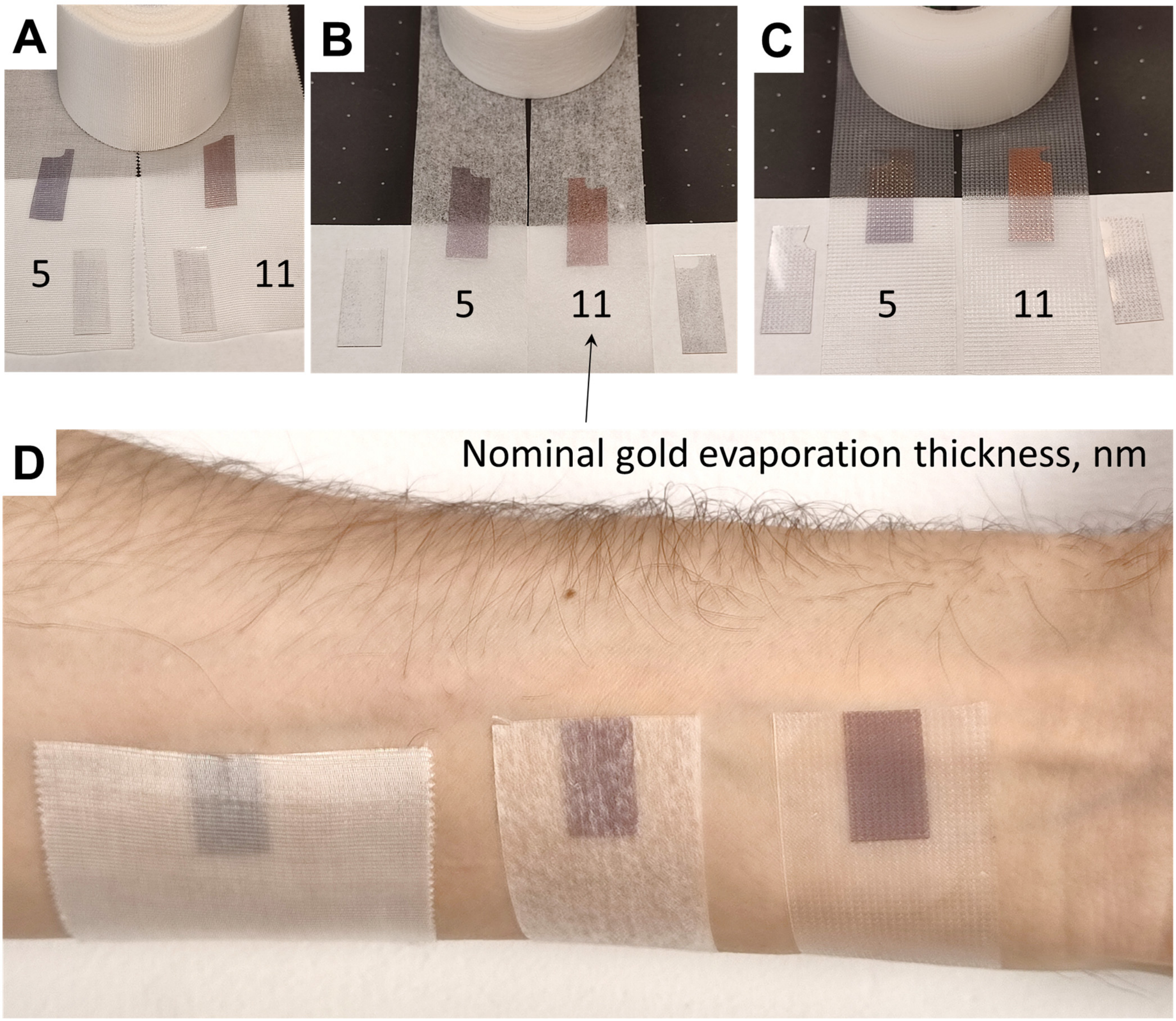

Figure S30. *Digital images of (**a**) 3M Durapore, (**b**) 3M Micropore, and (**c**) 3M Transpore medical tapes with transferred AuNIs and (**d**) the same tapes stuck to a hand.*

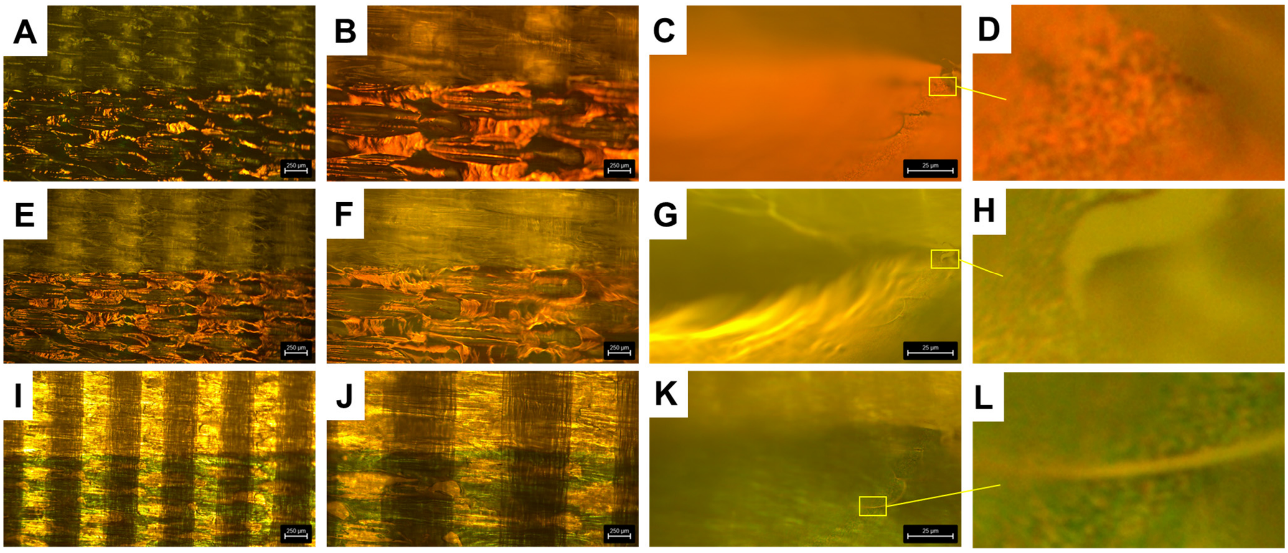

Figure S31. *Optical microscopy images on different scales and different illumination modes of 3M Durapore medical tape with a transferred AuNI (11 nm of nominal gold evaporation thickness) pattern:* ***(a-d)*** *– dark field mode,* ***(e-h)*** *– reflectance bright field mode, and* ***(i-l)*** *– transmittance mode.*

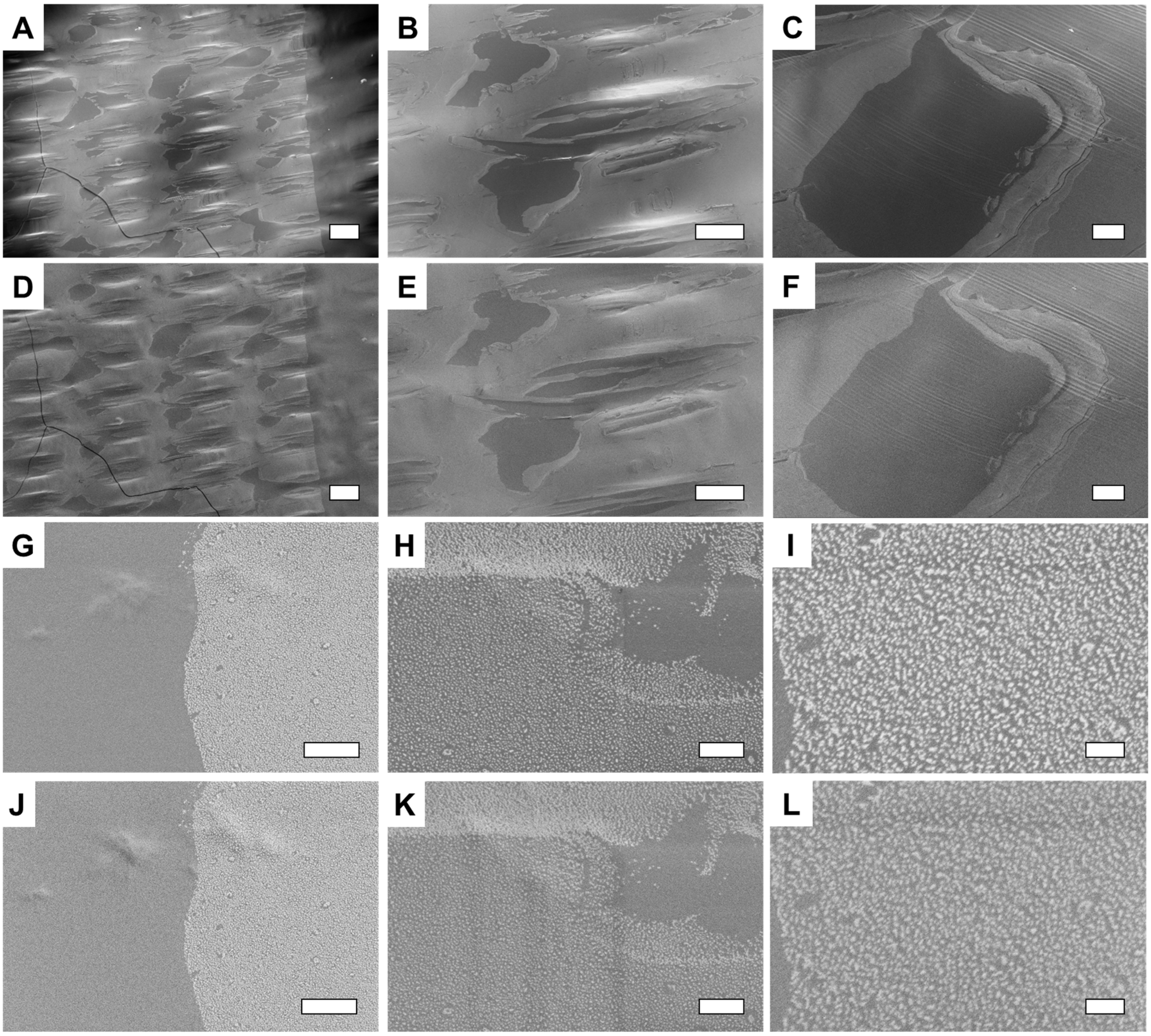

Figure S32. *FE-HRSEM images of 3M Durapore medical tape with transferred AuNIs of 5 nm of nominal gold evaporation thickness. Scalebars for (**a, d**) – 200 µm, (**b, e**) – 100 µm, (**c, f**) – 20 µm, (**g, j**) – 2 µm, (**h, k**) – 1 µm, and (**i, l**) – 400 nm. Images of (**a-c** and **g-i**) were taken from an in-lens detector, whereas images of (**d-f** and **j-l**) were taken from an SE2 detector.*

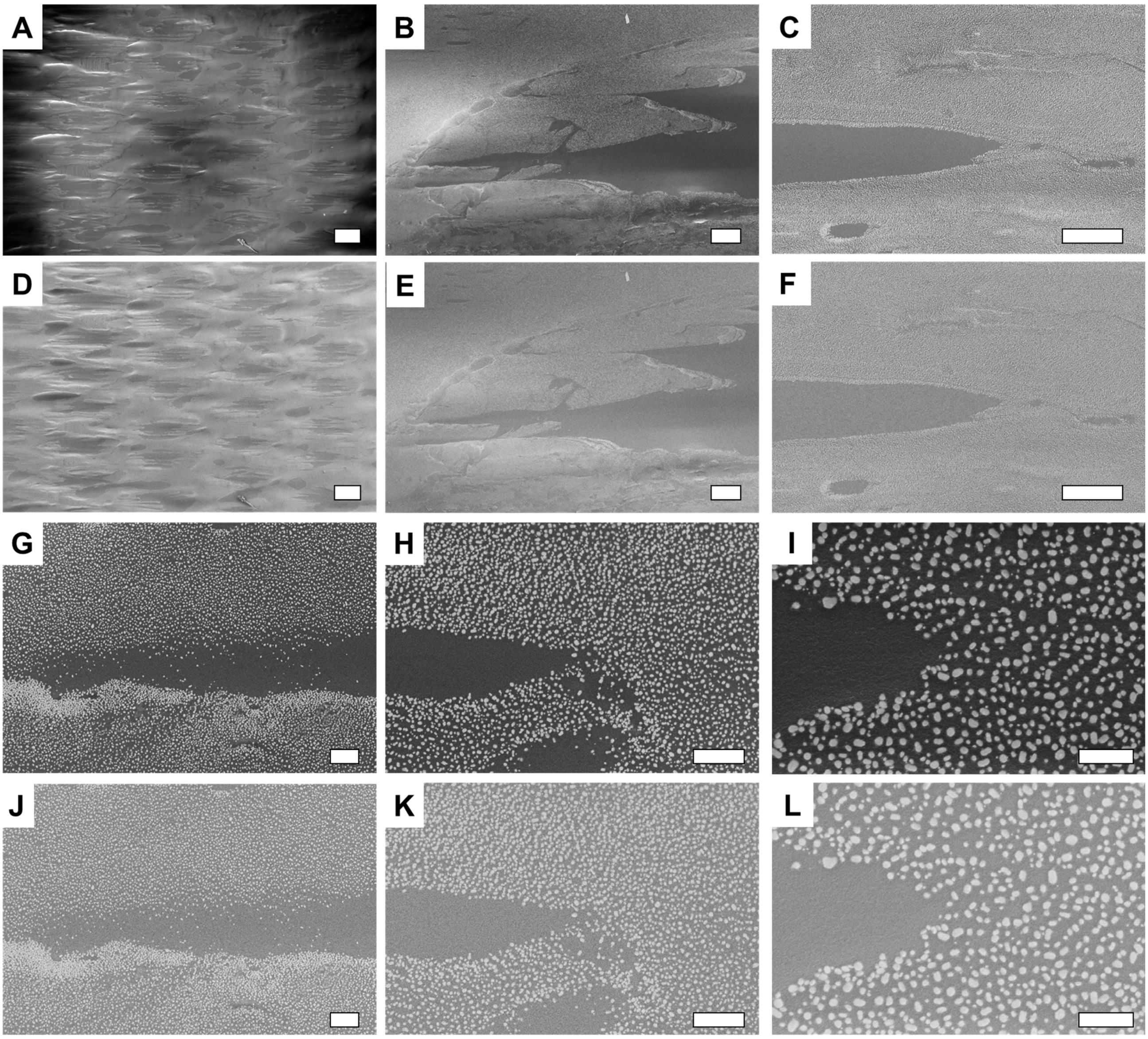

Figure S33. *FE-HRSEM images of 3M Durapore medical tape with transferred AuNIs of 11 nm of nominal gold evaporation thickness. Scalebars for **(a, d)** is 200 µm, **(b, e)** – 20 µm, **(c, f)** – 10 µm, **(g, h, j, k)** – 2 µm, and **(i, l)** – 1 µm. Images of **(a-c** and **g-i)** were taken from an in-lens detector, whereas images of **(d-f** and **j-l)** were taken from an SE2 detector.*

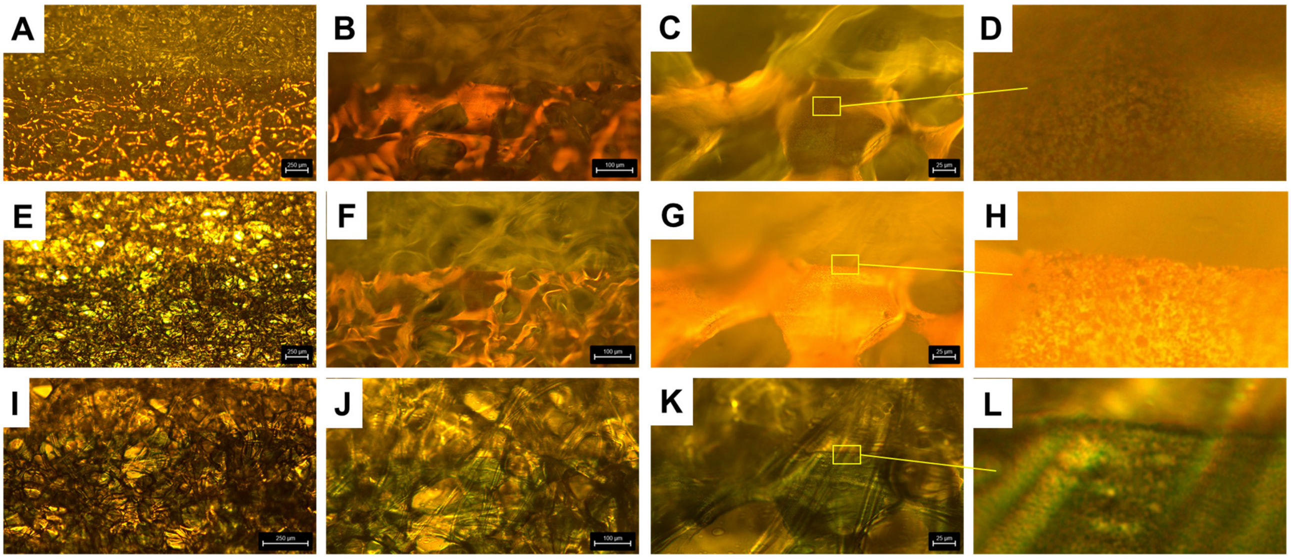

Figure S34. *Optical microscopy images at different scales and different illumination modes of 3M Micropore medical tape with a transferred AuNI (11 nm of nominal gold evaporation thickness) pattern: (**a-d**) – dark field mode, (**e-h**) – reflectance bright field mode, and (**i-l**) – transmittance mode.*

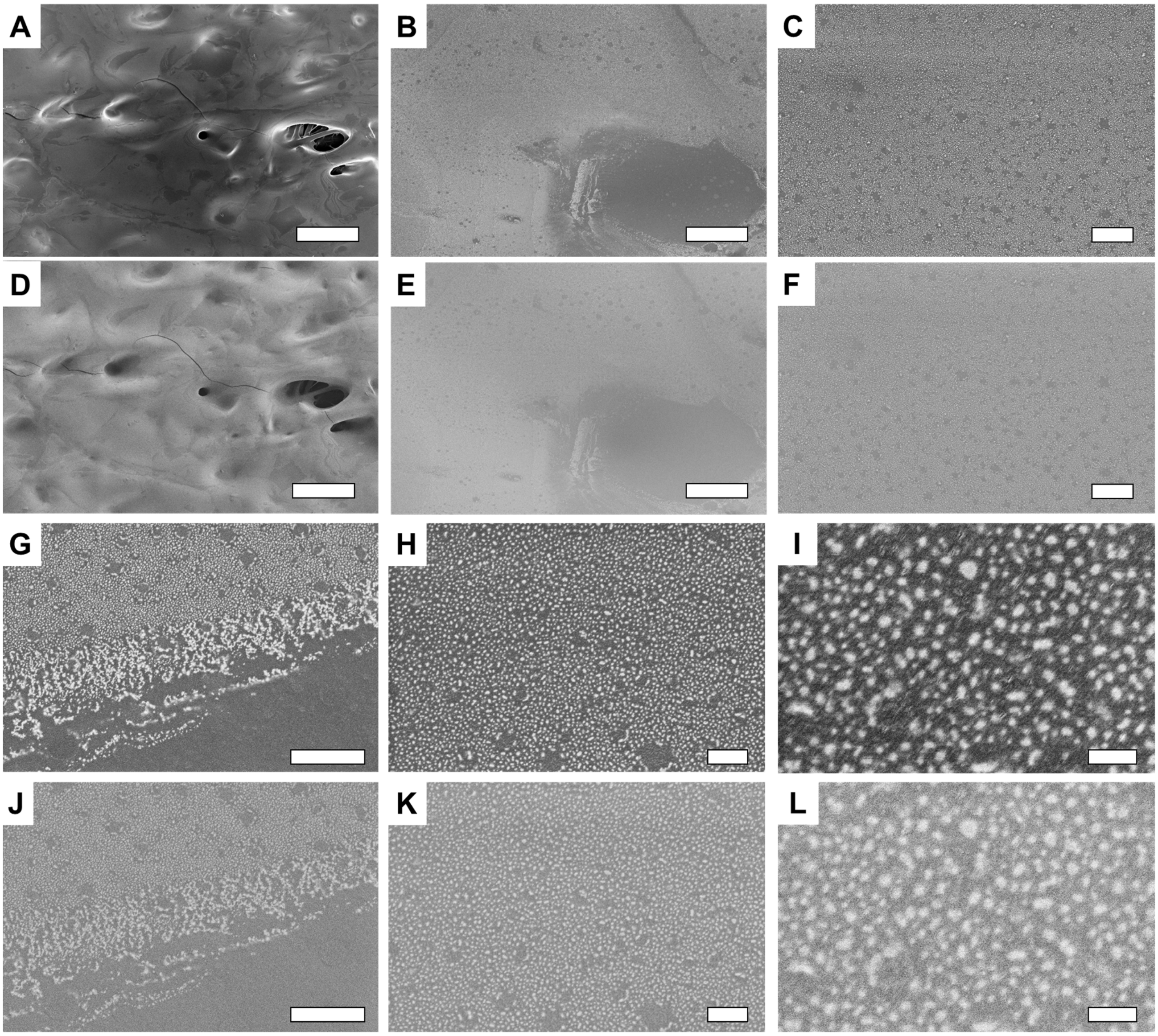

Figure S35. *FE-HRSEM images of 3M Micropore medical tape with transferred AuNIs with 5 nm of nominal gold evaporation thickness. Scalebar for (**a, d**) – 200 µm, (**b, e**) – 20 µm, (**c, f, g, j**) – 2 µm, (**h, k**) – 600 nm, (**i, l**) – 200 nm. Images of (**a-c** and **g-i**) were taken from in-lens detector while images of (**d-f** and **j-l**) were taken from SE2 detector.*

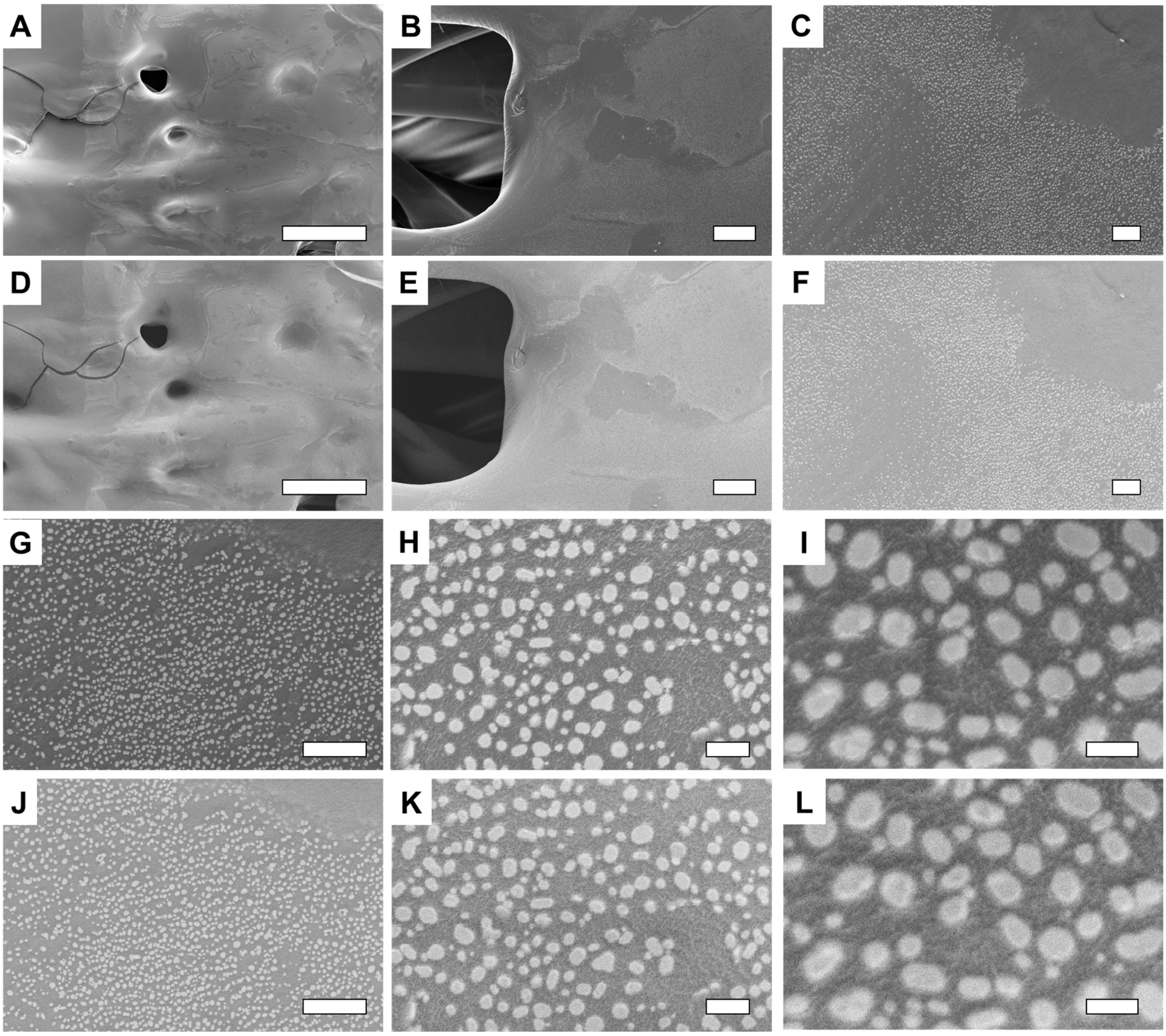

Figure S36. *FE-HRSEM images of 3M Micropore medical tape with transferred AuNIs of 11 nm of nominal gold evaporation thickness. Scalebars for **(a, d)** – 200 µm, **(b, e)** – 20 µm, **(c, f, g, j)** – 2 µm, **(h, k)** – 400 nm, and **(i, l)** – 200 nm. Images of **(a-c** and **g-i)** were taken from an in-lens detector, whereas images of **(d-f** and **j-l)** were taken from an SE2 detector.*

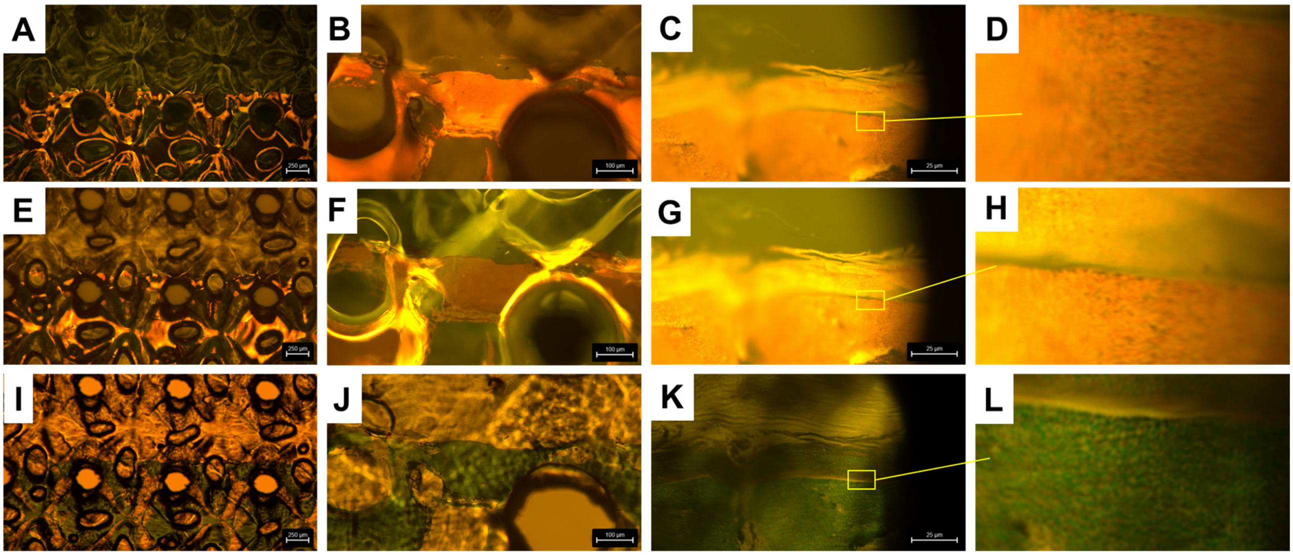

Figure S37. *Optical microscopy images at different scales and different illumination modes of 3M Transpore medical tape with a transferred AuNI (11 nm of nominal gold evaporation thickness) pattern:* ***(a-d)*** *– dark field mode,* ***(e-h)*** *– reflectance bright field mode, and* ***(i-l)*** *– transmittance mode.*

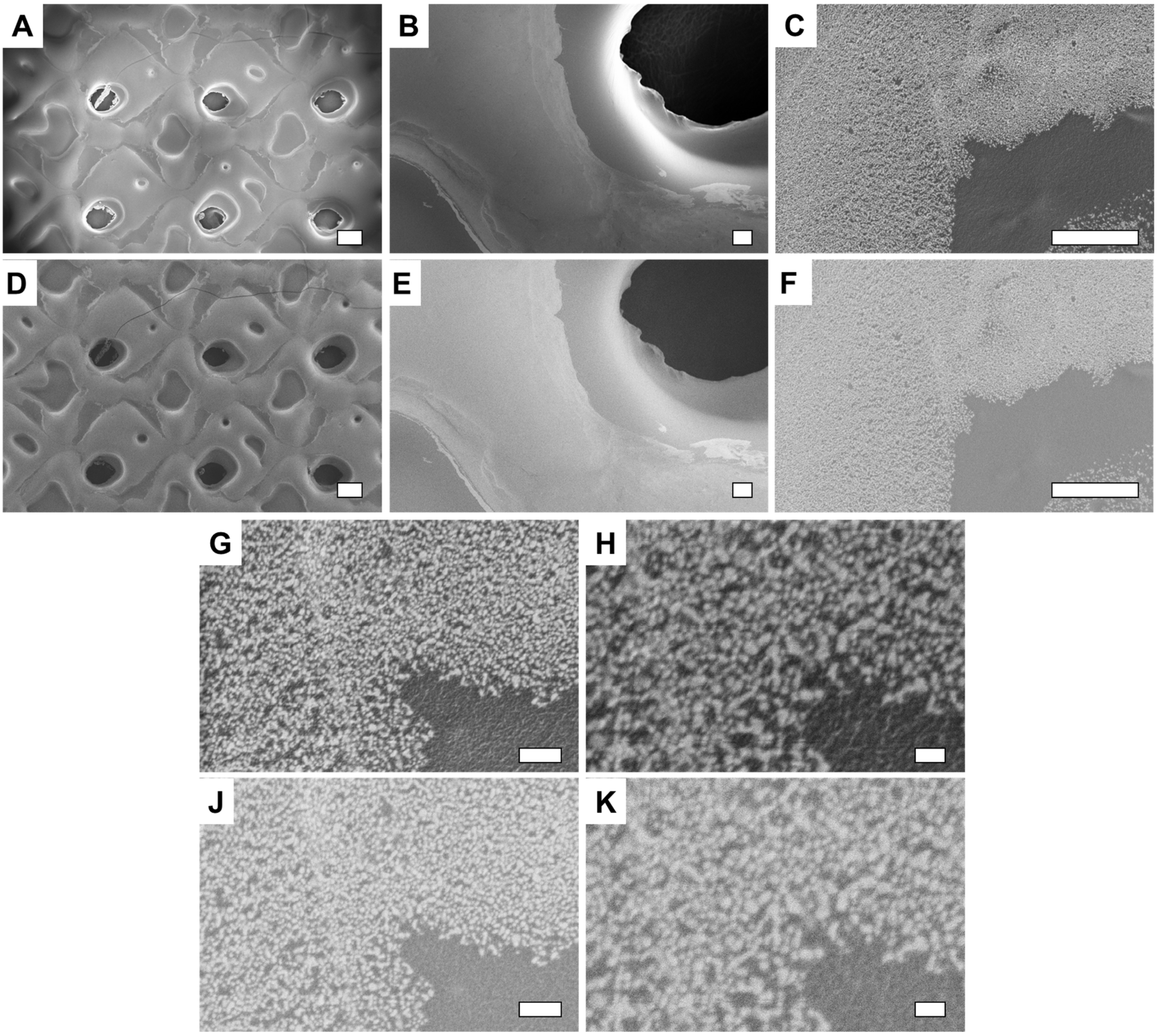

Figure S38. *FE-HRSEM images of 3M Transpore medical tape with transferred AuNIs of 5 nm of nominal gold evaporation thickness. Scalebars for (**a, d**) – 200 µm, (**b, e**) – 20 µm, (**c, f**) – 2 µm, (**g, j**) – 300 nm, and (**h, k**) – 100 nm. Images of (**a-c** and **g, h**) were taken from an in-lens detector, whereas images of (**d-f** and **j, k**) were taken from an SE2 detector.*

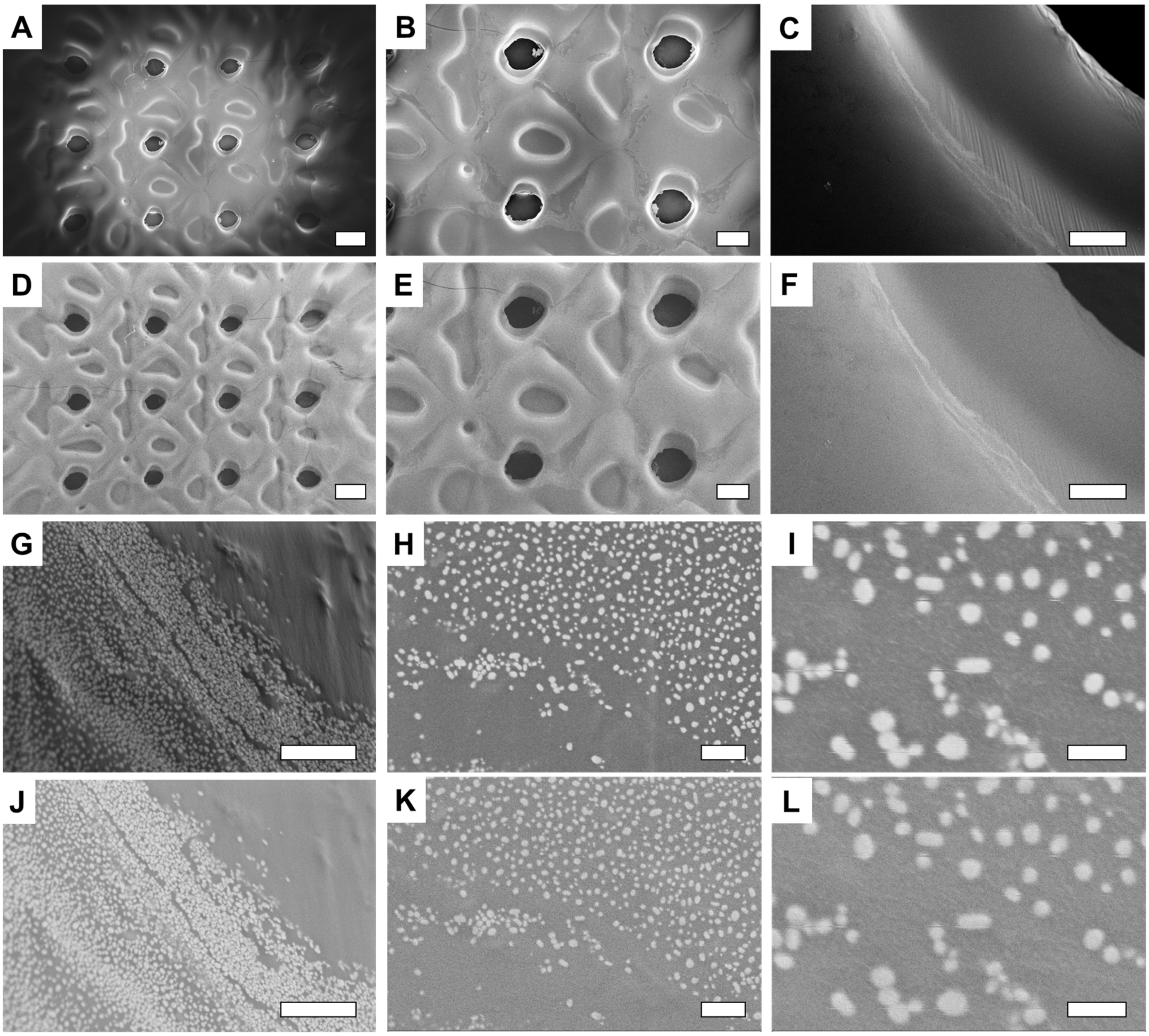

Figure S39. *FE-HRSEM images of 3M Transpore medical tape with transferred AuNIs of 11 nm of nominal gold evaporation thickness. Scalebars for* ***(a, d)*** *– 400 µm,* ***(b, e)*** *– 200 µm,* ***(c, f)*** *– 20 µm,* ***(g, j)*** *– 2 µm,* ***(h, k)*** *– 1 µm, and* ***(i, l)*** *– 400 nm. Images of* ***(a-c*** *and* ***g-i)*** *were taken from an in-lens detector, whereas images of* ***(d-f*** *and* ***j-l)*** *were taken from an SE2 detector.*

14. AuNIs transfer into other polymers

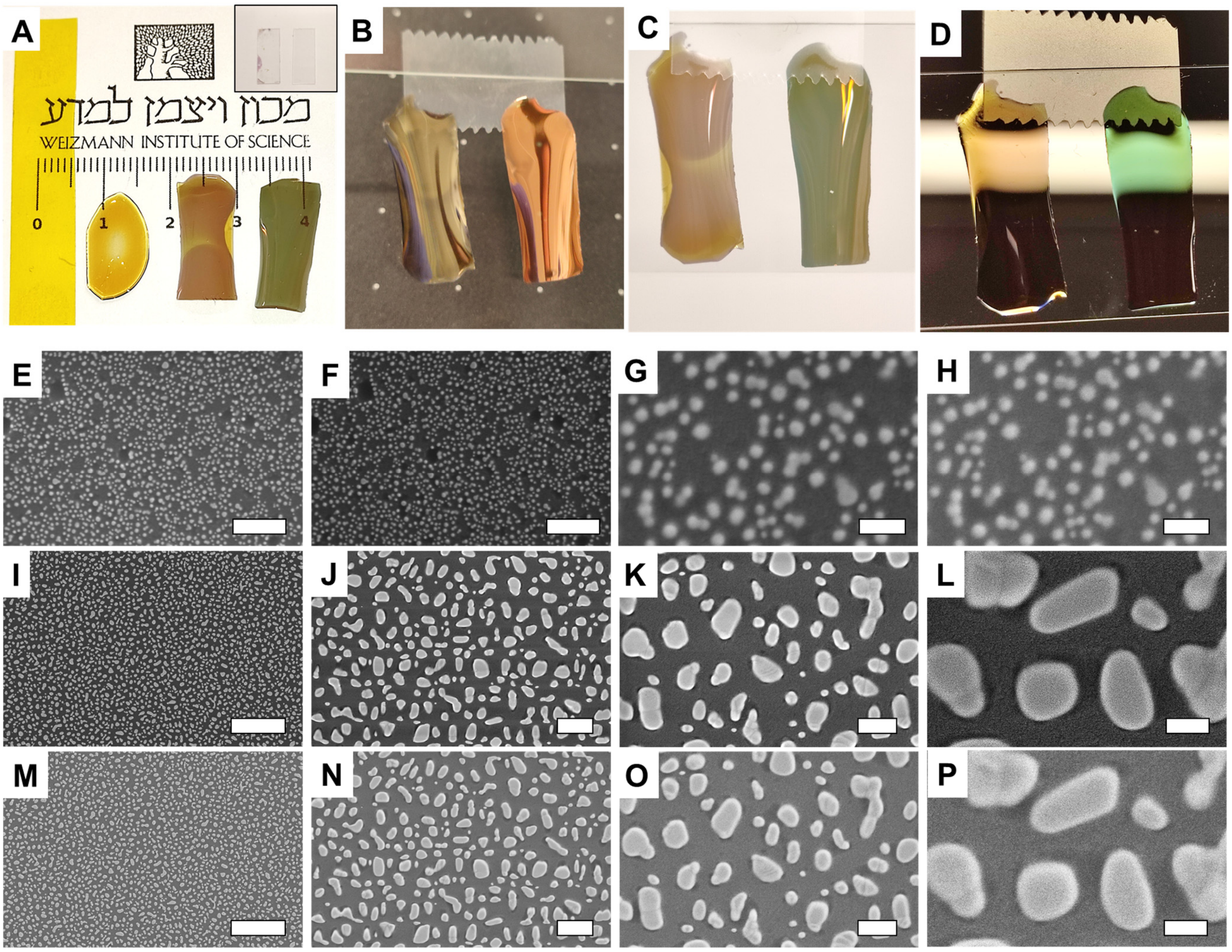

Figure S40. *Digital images in (**a**) reflectance mode on a white background of (from left to right): a Kapton tape, a drop of dried polyimide, a polyimide film doped with AuNIs of 5 and 11 nm (nominal evaporation thickness), image courtesy of the Weizmann Institute of Science. Inset of (**a**) – the corresponding glass slides after polyimide film separation. Polyimide films doped with AuNIs of 5 and 11 nm (nominal evaporation thickness) in (**b**) reflectance mode on a black background, (**c**) in transmittance mode on a white background and (**d**) in transmittance mode on a black background with backside illumination. (**e-p**) FE-HRSEM images of the polyimide films doped with AuNIs (nominal evaporation thickness) of (**e-h**) 5 nm and (**i-p**) 11 nm taken from (**e, g, i-l**) an in-lens detector and (**f, h, m-p**) from an SE2 detector at different magnifications. Scalebars: (**e, f, j, n**) – 400 nm, (**g, h, l, p**) – 80 nm, (**i, m**) – 2 µm, and (**k, o**) – 200 nm.*

15. COMSOL simulations of AuNIs embedding

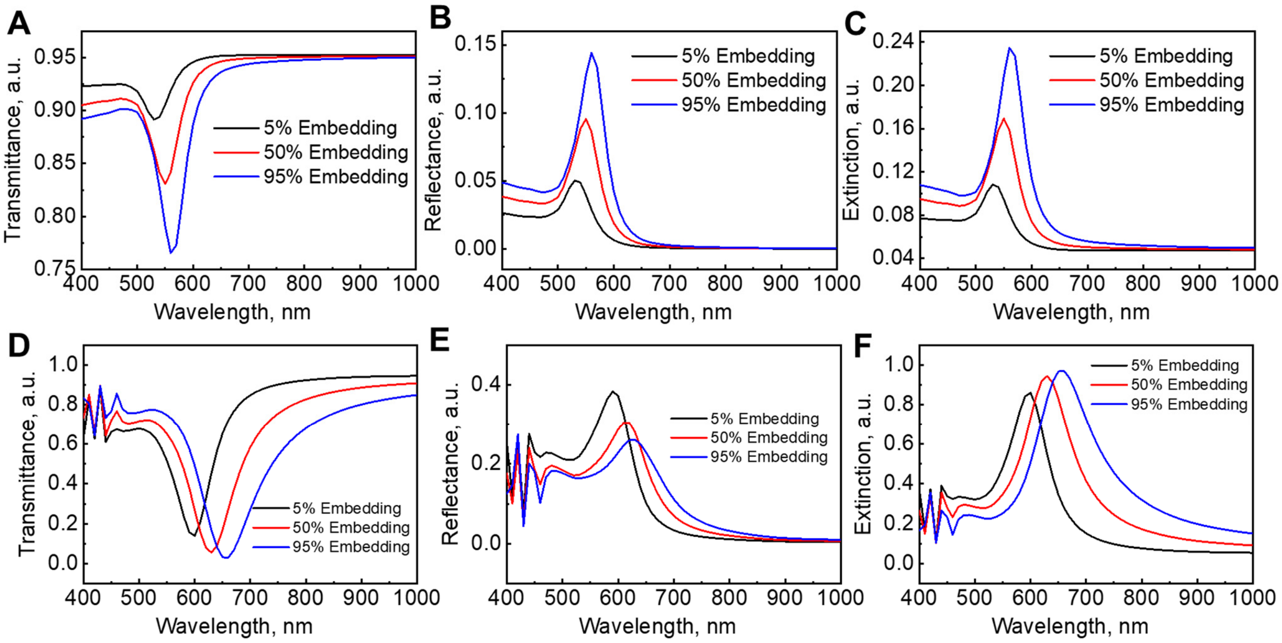

Figure S41. *Calculated normalized* ***(a, d)*** *transmittance,* ***(b, e)*** *reflectance, and* ***(c, f)*** *extinction (reflectance + absorbance) spectra of the AuNIs of* ***(a-c)*** *23 nm and* ***(d-f)*** *130 nm in diameter at different embedding values into a substrate (5, 50, and 95%).*

16. Contact angle measurements and surface hydrophobicity

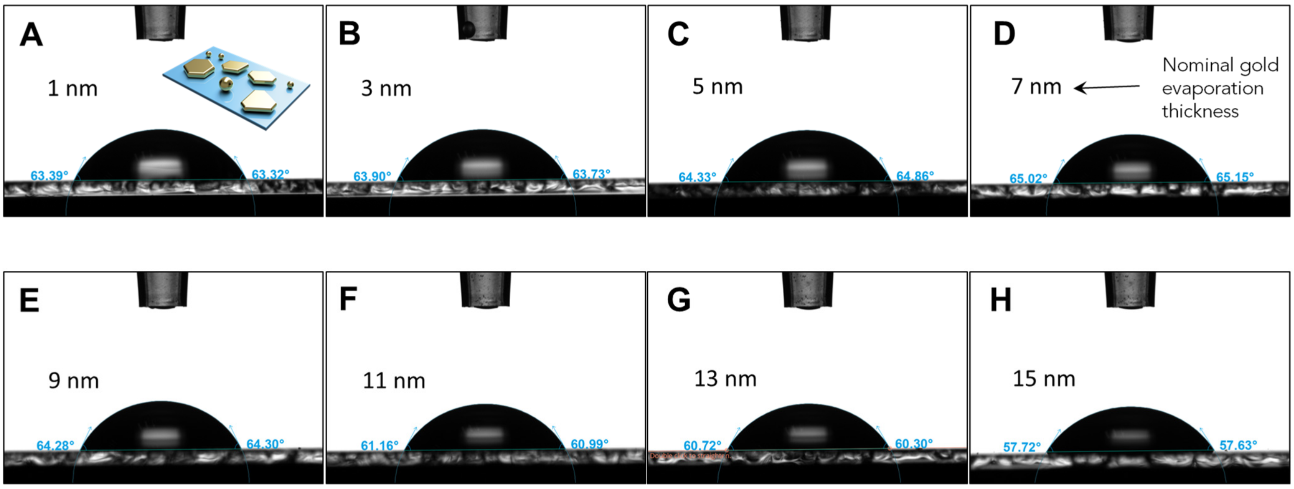

***Figure S42**. Contact angle measurements of a sessile drop (ca. 6 µl) of water on glass with AuNIs (nominal gold evaporation thickness: **a** – 1 nm, **b** – 3 nm, **c** – 5 nm, **d** – 7 nm, **e** – 9 nm, **f** – 11 nm, **g** – 13 nm, and **h** – 15 nm).*

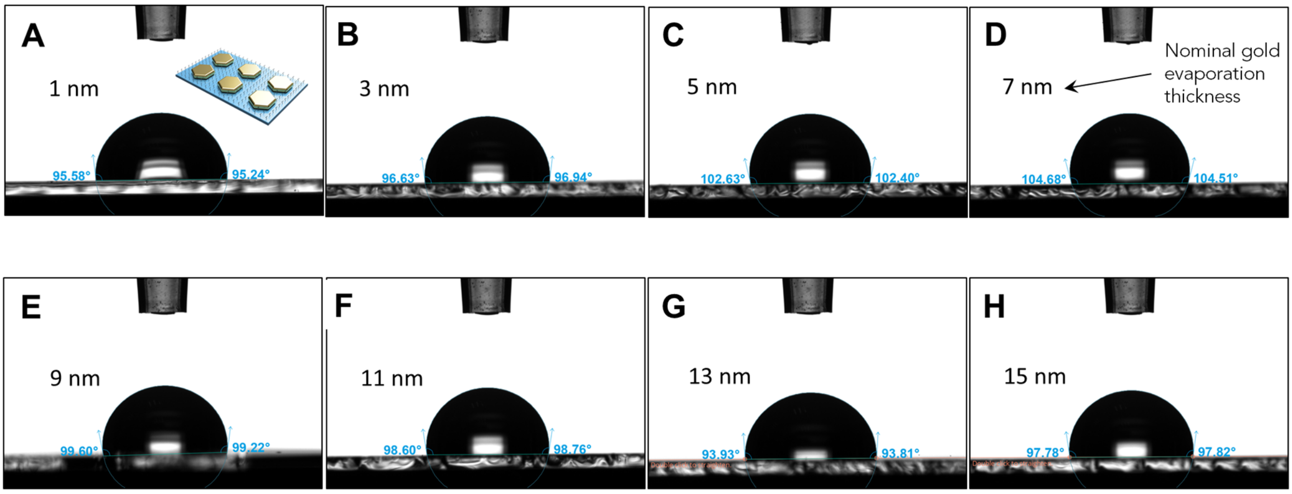

***Figure S43**. Contact angle measurements of a sessile drop (ca. 6 µl) of water on glass with AuNIs after silanization (nominal gold evaporation thickness: **a** – 1 nm, **b** – 3 nm, **c** – 5 nm, **d** – 7 nm, **e** – 9 nm, **f** – 11 nm, **g** – 13 nm, and **h** – 15 nm).*

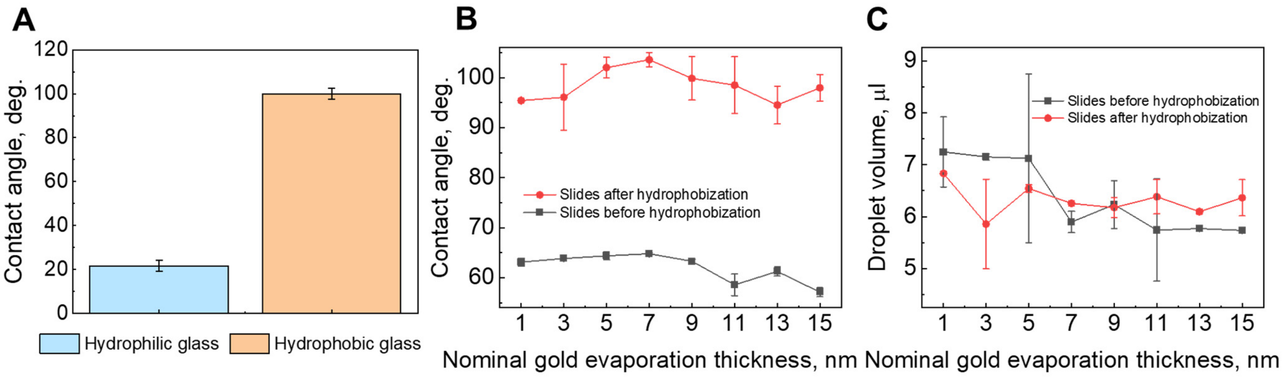

Contact angle, deg.	
Hydrophilic glass	21.7 ± 2.5
Hydrophobic glass	100.0 ± 2.6

Au nominal evaporation thickness, nm	Slides before hydrophobization	Slides after hydrophobization
1	63.1 ± 0.8	95.4 ± 0.1
3	63.9 ± 0.1	96.1 ± 6.6
5	64.4 ± 0.8	102.0 ± 2.1
7	64.8 ± 0.2	103.6 ± 1.4
9	63.3 ± 0.4	99.9 ± 4.3
11	58.5 ± 2.2	98.5 ± 5.7
13	61.3 ± 0.9	94.5 ± 3.8
15	57.1 ± 0.9	98.0 ± 2.7

Droplet volume, µl	
Hydrophilic glass	6.8 ± 0.1
Hydrophobic glass	5.8 ± 0.3

Au nominal evaporation thickness, nm	Slides before hydrophobization	Slides after hydrophobization
1	7.3 ± 0.7	6.8 ± 0.1
3	7.2 ± 0.1	5.9 ± 0.9
5	7.1 ± 1.6	6.5 ± 0.1
7	5.9 ± 0.2	6.3 ± 0.1
9	6.2 ± 0.5	6.2 ± 0.2
11	5.7 ± 1.0	6.4 ± 0.3
13	5.8 ± 0.1	6.1 ± 0.1
15	5.7 ± 0.1	6.4 ± 0.3

Figure S44. *Top panel: A summary of contact angle measurement statistics of a sessile drop of water on glass slides with and without AuNIs before and after silanization. (**a**) A comparison of the drop contact angle on the glass slide before and after silanization, (**b**) a comparison of the drop contact angle on the glass with AuNIs, before and after silanization, and (**c**) the corresponding statistics of the drop volume. Bottom panel: Summary tables for contact angles (left) and drop volume measurements (right).*

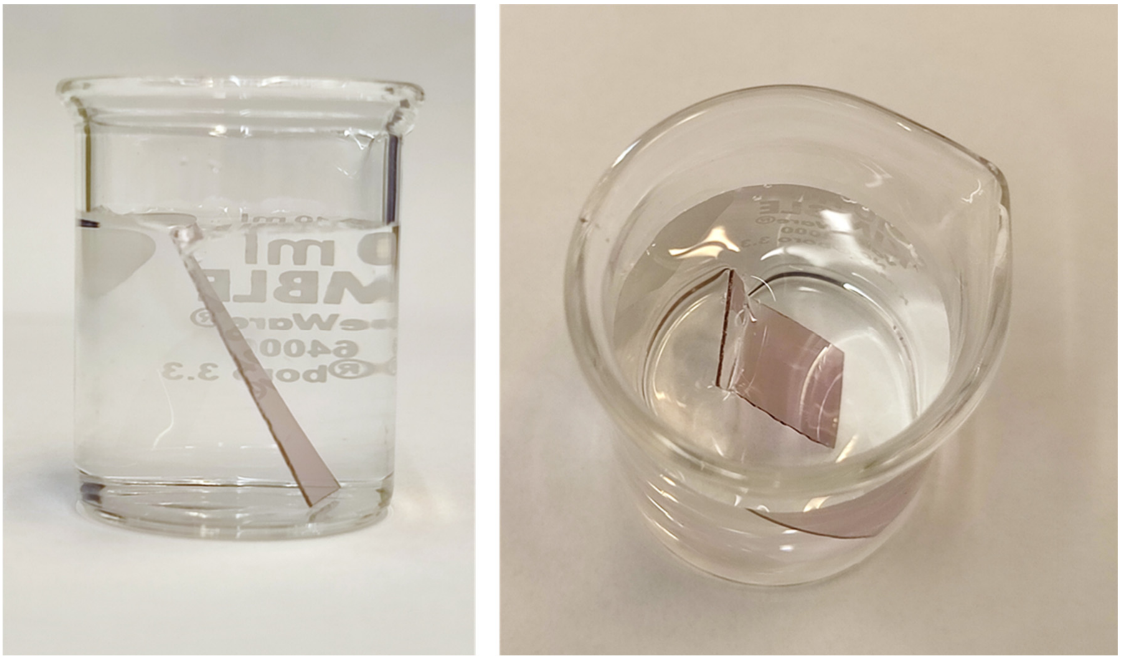

Figure S45. *Hydrophobic properties of a slide with AuNIs after silanization.*

The chemical vapor deposition (CVD) procedure of perfluorosilane was developed and employed on the AuNI-deposited glass substrates (see the experimental section, and **Figure S9**). Either triethoxy(1H,1H,2H,2H-perfluorooctyl)silane or trichloro(1H,1H,2H,2H-perfluorooctyl)silane can be used; both form a hydrophobic monolayer reacting with SiO_2. The former is a mild reagent, whereas the latter is more aggressive due to the release of HCl, formed as a byproduct of hydrolysis, which may slightly etch Au, yet is more efficient in terms of surface hydrophobicity (**Figure S10)**. A WCA of AuNI on glass slides before treatment was 63° – 65° and 57° – 63° for types I and II, respectively (**Figure S42, Figure S44)**, whereas after fluoro-silanization, all slides displayed increased hydrophobicity with WCAs in ranges between 95° and 104° (**Figure S43, Figure S44**). It is notable that no trend in WCA was spotted in terms of AuNI type and surface coverage. Prior to applying the polymer tape, the hydrophobization efficiency was also checked by immersing the slide into Milli-Q: modified glass readily repels water and may easily float in a beaker (**Figure S45**).

REFERENCES

(1) Karakouz, T.; Tesler, A. B.; Bendikov, T. A.; Vaskevich, A.; Rubinstein, I. Highly Stable Localized Plasmon Transducers Obtained by Thermal Embedding of Gold Island Films on Glass. *Adv. Mater.* **2008**, *20* (20), 3893–3899.

(2) Karakouz, T.; Tesler, A. B.; Sannomiya, T.; Feldman, Y.; Vaskevich, A.; Rubinstein, I. Mechanism of Morphology Transformation during Annealing of Nanostructured Gold Films on Glass. *Phys. Chem. Chem. Phys.* **2013**, *15* (13), 4656–4665.
